# Parameterized Complexity of Theory of Mind Reasoning in Dynamic Epistemic Logic

**DOI:** 10.1007/s10849-018-9268-4

**Published:** 2018-05-14

**Authors:** Iris van de Pol, Iris van Rooij, Jakub Szymanik

**Affiliations:** 1 Institute for Logic, Language and Computation, University of Amsterdam, Amsterdam, The Netherlands; 2 Donders Institute for Brain, Cognition, and Behaviour, Centre for Cognition, Radboud University, Nijmegen, The Netherlands

**Keywords:** Theory of mind, Higher-order reasoning, Computational-level analysis, Dynamic epistemic logic, Parameterized complexity, Intractability

## Abstract

Theory of mind refers to the human capacity for reasoning about others’ mental states based on observations of their actions and unfolding events. This type of reasoning is notorious in the cognitive science literature for its presumed computational intractability. A possible reason could be that it may involve higher-order thinking (e.g., ‘you believe that I believe that you believe’). To investigate this we formalize theory of mind reasoning as updating of beliefs about beliefs using dynamic epistemic logic, as this formalism allows to parameterize ‘order of thinking.’ We prove that theory of mind reasoning, so formalized, indeed is intractable (specifically, PSPACE-complete). Using parameterized complexity we prove, however, that the ‘order parameter’ is not a source of intractability. We furthermore consider a set of alternative parameters and investigate which of them *are* sources of intractability. We discuss the implications of these results for the understanding of theory of mind.

## Introduction

Imagine you are in love. You find yourself at your desk and you cannot stop your mind from wandering off. What is she thinking about right now? And more importantly, is she thinking about you, and does she know that you are thinking about her? You look at your phone, no message yet. Does this mean she is not interested, or is she waiting for you to send a message first? This kind of reasoning is an example of the cognitive capacity called ‘theory of mind,’^1^ which is the capacity for attributing mental states to oneself and others and to reason about these to explain behavior. This capacity is widely studied in various fields such as psychology, philosophy, biology, and cognitive (neuro)science (see, e.g., Frith 2001; Nichols and Stich 2003; Premack and Woodruff 1978; Verbrugge 2009; Wellman et al. 2001). What is especially interesting about the study of theory of mind is that researchers are puzzled by the heavy computational load that the use of theory of mind seems to imply (cf. Apperly 2011; Carruthers 2006; Haselager 1997; Levinson 2006; Sperber and Wilson 1996; Zawidzki 2013). Theory of mind seems to be so computationally demanding that successfully engaging in it seems impossible for resource-bounded minds like our own. As Ian Apperly describes:

[T]he problem is that an agent may have any number of beliefs (and other mental states), any of which might be relevant when trying to judge what the agent will think or do in a given situation. [...] [W]e should be extremely worried by the potentially intractable computational problems posed by mindreading. (Apperly 2011, p. 9, p. 179)

Although there is wide agreement that theory of mind poses a computationally intractable problem, precisely which aspects of theory of mind are sources of this intractability is highly debated (cf. Apperly 2011; Carruthers 2006; Haselager 1997; Levinson 2006; Slors 2012; Zawidzki 2013). One aspect that is recognized in the literature as potentially aggravating the computational load of theory of mind, is the use of higher-order thinking, such as the attribution of beliefs about beliefs. For instance, second-order belief attribution (such as, ‘Ayla thinks that Murat believes in unicorns’) already seems to be more difficult than first-order belief attribution (Flobbe et al. 2008; Hedden and Zhang 2002; Miller 2009; Wellman et al. 2001). Furthermore, several studies that investigated perspective taking at even higher levels found a prominent drop in performance from the fourth level (Kinderman et al. 1998; Lyons et al. 2010; Stiller and Dunbar 2007; but see also O’Grady et al. 2015). However, since most accounts of theory of mind—and the claims about their supposed (in)tractability—are expressed at an informal level, it is unclear whether this ‘order parameter’ is indeed a source of intractability and whether restricting it could render theory of mind tractable.

To investigate this we formalize theory of mind reasoning as updating of beliefs about beliefs using dynamic epistemic logic, as this formalism allows to parameterize the order of higher-order belief attribution. We prove that theory of mind reasoning, so formalized, indeed is intractable (specifically, PSPACE-complete). Using parameterized complexity we prove that, contrary to common belief, the order parameter (formalized as the modal depth of epistemic formulas) is not a source of intractability. We furthermore consider a set of alternative parameters and investigate which of them are sources of intractability. We thereby also provide a technical contribution with respect to dynamic epistemic logic (DEL)—we present fixed-parameter intractability and tractability results for the model checking problem for DEL (see Table 1 and Fig. 24 for an overview of the results). To our knowledge, this is the first parameterized complexity analysis for problems related to dynamic epistemic logic.

This work is meant as a showcase for how logic and complexity theory can be used to address questions in cognitive science (see also Cherniak 1990; Frixione 2001; Isaac et al. 2014; Levesque 1988). We address both cognitive scientists and logicians who are interested in theory of mind reasoning and its supposed intractability. We present this work as an example to logicians of how they can contribute to cognitive science, and an illustration to cognitive scientists of how these kinds of analyses can address questions about what makes theory of mind intractable or not. To this end, we include a primer on the conceptual background of computational-level modeling (Marr 1982), and the methodology of using complexity theory in cognitive science. Readers that are mainly interested in the implications of our results for the understanding of theory of mind may choose to skip the details of the complexity-theoretic results as these are not essential to understand the argument that we present. Furthermore, our complexity results may be of independent interest to researchers interested in the (parameterized) complexity of DEL model checking.

The paper is structured as follows. First, in Sect. 2, we introduce the framework of modeling cognitive capacities at Marr’s computational level and how and why to formalize the notion of complexity of theories of cognition. Then, in Sect. 3, we discuss the preliminaries of dynamic epistemic logic and introduce our formalism of updating of beliefs about beliefs as a form of model checking for dynamic epistemic logic. In Sect. 4, we discuss the preliminaries of (parameterized) complexity theory, and we analyze the classical and parameterized computational complexity of our formalization. In Sect. 5 we discuss the implications of these results for the understanding of theory of mind. Finally, in Sect. 6, we conclude and suggest directions for future research.

## Conceptual and Methodological Background

In this section, we explain the conceptual and methodological foundations of the approach that we use to analyze the computational complexity of theory of mind and its purported sources of intractability. This approach has been proposed, among others, by van Rooij and colleagues (van Rooij 2008; van Rooij and Wareham 2008, 2012) and builds on a combination of computational-level modeling on the one hand, and the use of concepts and proof techniques from classical and parameterized complexity theory on the other hand.

### Modeling at the Computational Level

To make formal claims about the complexity of theory of mind, we first need to define a model that captures theory of mind, or at least captures a necessary sub-capacity of theory of mind. This model should be a general model of the capacity, as opposed to a task-specific model of theory of mind in which not the capacity as such, but the application of it in a specific task is modeled. If we were to limit ourselves to a task-specific model, our results would hold for a specific instance of theory of mind but not necessarily generalise to other instances of theory of mind and therefore say little about the computational complexity of the general capacity.

There are several task-specific models of instances of theory of mind—both probabilistic and logic-based—mainly focussed on false-belief tasks. Braüner (2014), Bolander (2014), Stenning and van Lambalgen (2008), and Arkoudas and Bringsjord (2009) have presented different formalizations of several false-belief tasks, using respectively hybrid logic, dynamic epistemic logic, the event calculus, and closed-world reasoning. Arslan et al. (2013) defined an ACT-R model for the first and second-order false-belief task. Baker (2012) presented a Bayesian model for several alternative theory of mind tasks.

Blokpoel et al. (2013) and van Rooij et al. (2011) also proposed generic models of aspects of theory of mind, such as goal inference and recipient design, but these did not explicitly model the attribution of beliefs and beliefs about beliefs. Since our goal here is to specifically investigate the (parameterized) complexity of higher-order theory of mind we will propose a generic model based on dynamic epistemic logic. This formalism allows us to parameterize ‘order of thinking’ in a flexible way; i.e., there is no need to hard-code an upper limit on the order.

Besides the distinction between a generic and a task-specific model of a cognitive capacity, there are different levels of description at which a model can be pitched. The model that we put forth in this paper is situated at what Marr (1982) called the *computational level*. This is a description of a capacity in terms of what (kind of) problem needs to be solved, defined by a collection of constraints that need to be satisfied. This kind of description can be specified in terms of an input-output mapping, i.e., as a computational problem.

In addition, Marr defined two other levels of description, namely the *algorithmic level* and the *implementational level*. An algorithmic-level description defines the algorithm that is used to solve a problem, and it specifies the representations that the algorithm operates on. For a given computational-level description of a capacity, there can be many different algorithmic-level descriptions compatible with it, i.e., that compute the problem defined at the computional level. Lastly, an implementational-level description defines how an algorithm is implemented physically. Again it holds that the same algorithm can be implemented by different physical realizations.

When we present our results about the complexity of our formal description of theory of mind, we want our results to hold irrespective of the choice of a specific algorithm or implementation. That is why we define our formal description of theory of mind at Marr’s computational level.

### The Tractable Cognition Thesis

It is generally assumed among cognitive scientists that human cognitive capacities are constrained by computational tractability. This assumption is also known as the Tractable Cognition thesis (Frixione 2001; van Rooij 2008). The main ingredients of this thesis are (1) the observation that humans have limited cognitive resources, and (2) human cognitive capacities are confined to those that can be realized in a realistic amount of time, given the available cognitive recourses. To see that (1) is the case it is enough to realize that humans are finite beings in a finite surrounding and that our resources for cognitive processing are thereby bounded. Whether we see cognition as something that is done by means of our brain, the rest of our body, our environment, or by the interaction of these, these bounds remain. Clearly, any process that would involve more particles than there are present in the universe and millions of years to complete, would not be a plausible explanation of human cognition.

Intuitively we can understand the notion of intractability of models of cognition by looking at the goal of cognitive science and the notion of scalability. Cognitive science can be described as the study of human cognitive capacities, and one of its primary aims is to form theories that explain what these capacities are exactly, and how they work (Cummins 2000). The goal is to understand these capacities, taking into account the wide variety and richness of how we manifest them in real life. A common way to study these capacities is by means of experiments in which subjects are asked to perform a specific task. To meet scientific standards of controllable conditions and feasibility in lab settings, the situations that are presented in experiments are of much smaller scale than the wide range of situations that people face in their daily lives. So in practice these experiments deal with the specific application of a capacity in a toy domain, as a way to tap into the full-blown capacity. Given that humans have limited cognitive resources, a model of a cognitive capacity needs to be scalable in such a way that it can potentially also explain how we apply the capacity in real-world situations that are much more varied, open ended, and of larger size than the toy domains of experimental settings. An intuitive way of thinking about the (in)tractability of models of cognition is by this notion of scalability. When a model is intractable then it cannot plausibly scale up to provide explanations for the domain of real-life situations.

### The P-Cognition Thesis

For the tractable cognition thesis to be of optimal use, we of course want to be more formally precise about what it means for a model to be intractable. There have been several proposals for how to formalize this by using tools from computational complexity theory. In computational complexity theory the classical characterization of (in)tractability is done by the distinction between polynomial-time computability and NP-hardness (see, e.g., Garey and Johnson 1979). The class of computational problems that are polynomial-time solvable are seen as tractable, whereas problems that are NP-hard are considered intractable. If the commonly believed (yet unproven) claim that $\text { P}\ne \text { NP}$ holds, then NP-hard problems cannot be solved in polynomial time.^2^ The fastest known algorithms for NP-hard problems take more than polynomial time, e.g., exponential time. In the same way as we described for unscalable models of cognition, it holds for NP-hard problems that small sized inputs might very well be manageable. However, as their inputs grow bigger, the time needed to solve them will quickly blow up to astronomical proportions.

This classical definition of intractability has been the inspiration for the formalization of the Tractable Cognition thesis in the form of the P-cognition thesis. The P-cognition thesis states that cognitive capacities are confined to those that can be realized using at most a polynomial amount of time (Frixione 2001; van Rooij 2008). This means that when we characterize cognitive capacities in the form of a computational-level model, that we only consider those input-output mappings that are polynomial-time computable as potential plausible models of cognition, in terms of the cognitive recourses that are required. Note that this is not the same as saying that all polynomial-time computable models are plausible models of cognition—being polynomial-time computable is merely posed as a necessary condition, not as a sufficient condition.

### The FPT-Cognition Thesis

An objection that has been posed to the P-cognition thesis by van Rooij (2008) is that the P-cognition thesis is too restrictive and risks rejecting potentially plausible models of cognition. When a computational problem is NP-hard for a certain input domain, this means that there is no algorithm that can solve the problem in polynomial time for all inputs in that domain. It could however be that when the input domain is restricted to inputs with a certain structure, the problem becomes much easier to solve. An example of this is the Traveling Salesperson Problem. The input to this problem is a set of cities, distances between them, a point of departure, and a maximum route length. The question that is asked is whether there is a route that starts and ends in the point of departure, visits all other cities exactly once, and does not exceed the maximum route length. This problem is known to be NP-hard when allowing all possible inputs to the problem (Karp 1972). However, when the cities are aligned exactly on a circle, the problem becomes almost trivial to solve. Even when not all cities are aligned on a circle but many of them are and those that are not lay inside this circle, the problem is still relatively easy to solve. This property of cities being close to aligned on a circle can be captured by drawing a line around the outermost cities (more formally, drawing the convex hull) and looking at the cities that lay in the interior of this hull. The fewer cities there are inside the hull, the easier the problem becomes to solve.

This notion of problems being easier to solve for inputs with a certain structure is captured by the computational complexity notion of fixed-parameter tractability. This notion is a central tool within parameterized complexity theory, a branch of complexity theory where complexity is measured not only in terms of the input size, but also in terms of an additional parameter (see, e.g., Downey and Fellows 2013). This parameter can be defined in such a way that it captures a particular type of structure in an input, where a low value of the parameter signifies that an input is highly structured, and a high value of the parameter means that an input has little structure. The Traveling Salesperson Problem, for instance, has been proven to be fixed-parameter tractable when parameterized by the number of interior points in the convex hull (Deineko et al. 2006).

This parameterized notion of tractability was the inspiration for the FPT-cognition thesis by van Rooij (2008), which states that cognitive capacities are confined to those that can be realized using at most a fixed-parameter amount of time for one or more input parameters that are small in practice. Since all models that are polynomial-time solvable are also fixed-parameter tractable, this means that the FPT-cognition thesis is a relaxed version of the P-cognition thesis. It allows for all models that were recognized by the P-cognition thesis as being tractable, and in addition it allows some more models that were not recognized by the P-cognition thesis as being tractable.

NP-hard models of cognition pervade cognitive science, spanning many different cognitive domains, such as vision (Tsotsos 1990; van Rooij 2003), reasoning (Oaksford and Chater 1998; Levesque 1988; Reiter 1980), planning (Bylander 1994; Newell and Simon 1988), language (Barton et al. 1987; Ristad 1993; Szymanik 2016; Wareham 1999) and decision-making (Otworowska et al. 2017; van Rooij et al. 2005). Under the P-cognition thesis it would be a natural response to disregard those models and scrape them from the list of plausible models. Such a practice would most likely lead to throwing out the baby with the bathwater. Models of cognition are often built on and informed by years of (experimental) research. When a model is NP-hard this does not mean that all parts of it are false or useless, on the contrary. An NP-hard model of cognition might be heading exactly in the right direction, capturing much of how a certain capacity really works. It could be the case that the intractability of that model is a result of overgeneralisation (see van Rooij 2015). This would mean that the model captures a much broader phenomenon, which might include capacities that people actually (can) perform but in addition also includes more general capacities that people do not and cannot perform. Fixed-parameter tractability can be a useful tool to explore how a model is overly general, and to identify structural properties related to the capacity of interest, that could be exploited to make a certain problem tractable. These ideas will be made precise in Sect. 4, after introducing our model of theory of mind. In our analysis of the complexity of theory of mind and in the interpretation of our results, we use the FPT-cognition thesis as our theoretical framework.

## Formalizing Theory of Mind Using Dynamic Epistemic Logic

In this section we present a computational-level model of a subcapacity of theory of mind, namely the updating of beliefs about beliefs in dynamic epistemic logic. First, we introduce some basic concepts and definitions of dynamic epistemic logic. Then, we give both an intuitive description and a formal definition of the model, and we illustrate this with an example.

### Preliminaries: Dynamic Epistemic Logic

Dynamic epistemic logic (DEL) is a particular kind of modal logic (see van Benthem 2011; van Ditmarsch et al. 2008), where the modal operators are interpreted in terms of belief or knowledge. For the reader that is familiar with the details of DEL it suffices to know that we use the same framework as van Ditmarsch et al. (2008) with two modifications. Following Bolander and Andersen (2011), we allow both single and multi-pointed models, and we include postconditions in our event models (which are mappings to propositional literals). The postconditions allow modeling ontic, i.e., factual, change, in addition to epistemic change—which we believe is needed for a general applicability of the model. Furthermore the use of multi-pointed models allow for representing the internal perspective of an observer (cf. Aucher 2010; Dégremont et al. 2014; Gierasimczuk and Szymanik 2011), instead of the perfect external view (i.e., the omniscient god perspective). For the purpose of this paper we focus mainly on epistemic models and event models as semantic objects and less on the corresponding language.

First, we define epistemic models, which are Kripke models with an accessibility relation for every agent $a \in \mathcal {A}$. Epistemic models are used to represent facts and beliefs in a given situation. Intuitively, an epistemic model represents how agents perceive a given situation: their knowledge, beliefs, or uncertainty about the facts and (the knowledge, beliefs, or uncertainty of) the other agents in that situation.

#### Definition 1

(*Epistemic model*) Given a finite set $\mathcal {A}$ of agents and a finite set $\text { P}$ of propositional variables, an *epistemic model* is a tuple (*W*, *R*, *V*), where:

- *W* is a non-empty set of worlds;
- *R* is a function that assigns to every agent $a \in \mathcal {A}$ a binary relation $R_a$ on *W*; and
- *V* is a valuation function from $W \times P$ into $\{0,1\}$.

The accessibility relations $R_a$ can be read as follows: for worlds $w,v \in W$, $w R_a v$ means ‘in world *w*, agent *a* considers world *v* possible.’

#### Definition 2

(*Pointed epistemic model*) A pair $(\mathcal {M}, W_d)$ consisting of an epistemic model $\mathcal {M}=(W,R,V)$ and a non-empty set $W_d \subseteq W$ of designated worlds is called a *pointed epistemic model*. A pair $(\mathcal {M}, W_d)$ is called a single-pointed model when $W_d$ is a singleton and a multi-pointed epistemic model when $|W_d| > 1$. By a slight abuse of notation, for $(\mathcal {M}, \{w\})$, we also write ($\mathcal {M},w$).

We consider the usual restrictions on relations in epistemic models and event models, such as KD45 and S5 relations (see van Ditmarsch et al. 2008). In KD45 models, all relations are transitive, Euclidean, and serial, and in S5 models all relations are transitive, reflexive, and symmetric.

We define the following language for epistemic models. We use the modal belief operator *B*, where for each agent $a \in \mathcal {A}$, $B_a \varphi$ is interpreted as ‘agent *a* believes (that) $\varphi$.’

#### Definition 3

(*Epistemic language*) The language $\mathcal {L}_B$ over $\mathcal {A}$ and *P* is given by the following definition, where *a* ranges over $\mathcal {A}$ and *p* over *P*:

$$\begin{aligned} \varphi : = p \ | \ \lnot \varphi \ | \ (\varphi \wedge \varphi ) \ | \ B_a \varphi . \end{aligned}$$

We will use the following standard abbreviations, $\top := p \vee \lnot p, \bot := \lnot \top , \varphi \vee \psi := \lnot (\lnot \varphi \wedge \lnot \psi )$, $\varphi \rightarrow \psi := \lnot \varphi \vee \psi$, $\hat{B_a} \varphi := \lnot B_a \lnot \varphi$.

The semantics for this language is defined as follows.

#### Definition 4

(*Truth in a single-pointed epistemic model*) Let $\mathcal {M}=(W,R,V)$ be an epistemic model, $w \in W$, $a \in \mathcal {A}$, and $\varphi , \psi \in \mathcal {L}_B$. We define $\mathcal {M}, w \models \varphi$ inductively as follows:

$$\begin{aligned} \begin{array}{lll} \mathcal {M},w \models p &{}\qquad \hbox { iff }&{}\qquad V(w,p)=1;\\ \mathcal {M},w \models \lnot \varphi &{}\qquad \hbox { iff }&{}\qquad \hbox { not }\mathcal {M}, w \models \varphi ;\\ \mathcal {M},w \models (\varphi \wedge \psi ) &{}\qquad \hbox { iff }&{}\qquad \mathcal {M}, w \models \varphi \hbox { and } \mathcal {M}, w \models \psi ; \hbox { and } \\ \mathcal {M},w \models B_a \varphi &{}\qquad \hbox { iff }&{}\qquad \hbox { for all } v \hbox { with } wR_av: \mathcal {M}, v \models \varphi .\\ \end{array} \end{aligned}$$

When $\mathcal {M},w \models \varphi$, we say that $\varphi$ is true in *w* or $\varphi$ is *satisfied* in *w*.

#### Definition 5

(*Truth in a multi-pointed epistemic model*) Let $(\mathcal {M},W_d)$ be a multi-pointed epistemic model, $a \in \mathcal {A}$, and $\varphi \in \mathcal {L}_B$. $\mathcal {M}, W_d \models \varphi$ is defined as follows:

$$\begin{aligned} \mathcal {M},W_d \models \varphi \hbox { iff } \mathcal {M},w \models \varphi \hbox { for all } w \in W_d. \end{aligned}$$

Next, we define event models. Event models represent changes brought about in some initial situation by actions or events and how these changes are perceived by agents, i.e., what these agents consider to have occurred and how this influences their beliefs. Such actions or events may be as broad as agents reading, speaking, hearing, or seeing, or physical changes in the environment. Event models are constructed very similarly to epistemic models. Intuitively, the events in an event model represent the possible events that are considered by the agents, and the relations between these events represent the knowledge, beliefs, or uncertainty of the agents about the actual event that has taken place.

#### Definition 6

(*Event model*) An *event model* is a tuple $\mathcal {E} = (E, Q, {{\textsf {{pre}}}}{}, {{\textsf {{post}}}}{})$, where *E* is a non-empty finite set of events; *Q* is a function that assigns to every agent $a \in \mathcal {A}$ a binary relation $Q_a$ on *E*; pre is a function from *E* to $\mathcal {L}_B$ that assigns to each event a precondition, which can be any formula in $\mathcal {L}_B$; and post is a function from *E* to $\mathcal {L}_B$ that assigns to each event a postcondition. Postconditions are conjunctions of literals: propositional variables and their negations (including $\top$).

The accessibility relation $Q_a$ can be read as follows: for events $e,f \in E$: $e Q_a f$ means ‘in event *e*, agent *a* considers event *f* possible.’

#### Definition 7

(*Pointed event model / action*) A pair $(\mathcal {E}, E_d)$ consisting of an event model $\mathcal {E}=(E,Q,{{\textsf {{pre}}}}{},{{\textsf {{post}}}}{})$ and a non-empty set $E_d \subseteq E$ of designated events is called a *pointed event model*. A pair $(\mathcal {E}, E_d)$ is called a single-pointed event model when $E_d$ is a singleton and a multi-pointed event model when $|E_d| > 1$. We will use the term “action” interchangeably with the term “event model”.

We define the notion of a product update, which is used to update epistemic models with event models (Baltag et al. 1998). Updating a pointed epistemic model with an (applicable) event model yields a new pointed epistemic model. Updating an epistemic model with an event model can result in a change in the domain, the relations, and/or the valuation of an epistemic model. The resulting epistemic model represents how the facts in the initial situation changed and how the agents updated their beliefs by the occurrence of the event.

#### Definition 8

(*Product update*) The *product update* of the state $(\mathcal {M}, W_d)$ with the action $(\mathcal {E}, E_d)$ is defined as $(\mathcal {M}, W_d) \otimes (\mathcal {E}, E_d) = ((W', R', V'), W'_d)$, where:

- $$W' = \{(w,e) \in W \times E\ ;\ \mathcal {M},w \models \text {pre}(e)\}$$;
- $$R'_a = \{((w,e), (v,f)) \in W' \times W' \ ; \ wR_av \text { and } eQ_af\}$$;
- $V'((w,e),p) = 1$ iff $(\mathcal {M},w \models p$ and $\lnot p \notin {{\textsf {{post}}}}{}(e) )$ or $p \in {{\textsf {{post}}}}{}(e)$; and
- $$W'_d = \{(w,e) \in W' \ ;\ w \in W_d \text { and } e \in E_d\}$$.

Finally, we define when event models are applicable in a state.

#### Definition 9

(*Applicability*) We say that an event model or action $(\mathcal {E}, E_d)$ is *applicable* in state $(\mathcal {M},W_d)$ if there is some $e \in E_d$ and some $w \in W_d$ such that $\mathcal {M},w \models pre(e)$. We define applicability for a sequence of event models or actions inductively. The empty sequence, consisting of no actions, is always applicable. A sequence $a_1, \ldots , a_k$ of actions is applicable in a pointed epistemic model $(M,W_d)$ if (1) the sequence $a_1, \ldots , a_{k-1}$ is applicable in $(M,W_d)$, and (2) the action $a_k$ is applicable in $(M,W_d) \otimes a_1 \otimes \cdots \otimes a_{k-1}$.

**Fig. 1:**
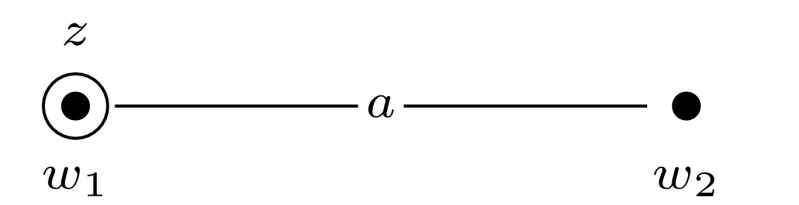
The epistemic model $(\mathcal {M},w_1)$ for the set $\mathcal {A}= \{a, b \}$ of agents and a single propositional variable *z*, where $\mathcal {M}= (W,R,V)$, $W = \{w_1,w_2 \}$, $R_a = \{(w_1,w_1), (w_1,w_2), (w_2,w_1), (w_2,w_2) \}$, $R_b = \{(w_1,w_1), (w_2,w_2) \}$, and $V(w_1, z) = 1$ and $V(w_2, z) = 0$

**Fig. 2:**
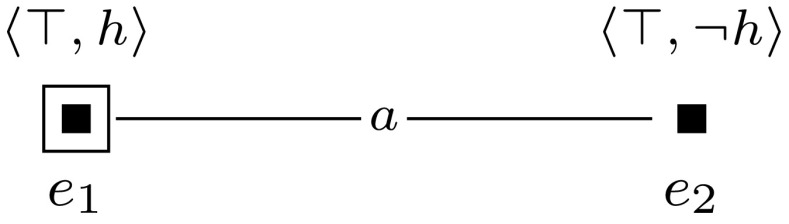
The event model $(\mathcal {E},e_1)$ for the set $\mathcal {A}= \{a, b \}$ of agents and a single propositional variable *h*, where $\mathcal {M}= (E,S,{\textsf {pre}},{\textsf {post}})$, $E = \{e_1,e_2 \}$, $R_a = \{(e_1,e_1), (e_1,e_2), (e_2,e_1), (e_2,e_2) \}$, $R_b = \{(e_1,e_1), (e_2,e_2) \}$, ${\textsf {pre}}(e_1) = {\textsf {pre}}(e_2) = \top$, ${\textsf {post}}(e_1) = h$, and ${\textsf {post}}(e_2) = \lnot h$

For convenience, we will depict epistemic models and event models graphically. We represent worlds with solid dots and events with solid squares, and we indicate the designated world(s) or event(s) with a circle or a square around that world or event. We represent the relations between worlds or events with lines that are labeled with the relevant agent symbols. Valuations are represented as labels next to the worlds: we label only those propositional variables that are true in a given world. Events are labeled with a tuple $\langle {{\textsf {{pre}}}}{}, {{\textsf {{post}}}}{}\rangle$: the first element of the tuple is the precondition and the second element is the postcondition of the event. When an element of the tuple is $\top$, this means that the event has no precondition (or postcondition). See Fig. 1 for an example of an epistemic model and Fig. 2 for an example of an event model. All epistemic models and event models that we present in our proofs are S5 models, i.e., they have equivalence relations. For the sake of presentation, in all our drawings we replace each relation $R_a$ with some $R_a'$, whose transitive reflexive closure is equal to $R_a$. For instance, each world has a reflexive relation to itself for every agent, which we omit.

### Dynamic Belief Update

Next, we present a computational-level model of a central sub-capacity of theory of mind reasoning, namely the updating of beliefs about beliefs, based on observed behavior and other factors of change. Our aim is to capture, in a qualitative way, the kind of reasoning that is necessary to be able to engage in (higher-order) theory of mind reasoning. We model this using dynamic epistemic logic (DEL), which allows us to parameterize higher-order reasoning. Specifically, we use a special case of the model checking problem for DEL. Complexity results (hardness) for this sub-capacity will hold (as a lower-bound) for the complexity of the full capacity. We present the model in the form of a decision problem, so that we can analyze its computational complexity using hardness-tools from computational complexity theory. A defining feature of theory of mind reasoning is the fact that it can vary in order. ‘Ahmed believes that I will pick him up at eight,’ is an example of first-order belief attribution. ‘Trish thinks that Fernando knows that we will throw him a surprise party,’ is an instance of second-order belief attribution. We formalize this order of theory of mind as the modal depth of epistemic formulas since a first-order belief attribution may be formalized by some sentence $B_a p$ with modal depth one and a second-order belief attribution by some sentence $B_a B_b p$ with modal depth two, and so on.

Intuitively, the following happens in the model. First, there is an initial situation in which a subject observes one or more agents. This subject has certain beliefs about the epistemic states and the (worldy) facts in this initial situation. Then, the subject observes some actions or events that bring about certain factual or epistemic changes. Based on this, the subject updates their beliefs about the epistemic states and the facts in the new situation.

The input of the model consists of the initial situation and the sequence of actions and events that happen, together with a question about the new situation. For instance, ‘does agent X believe Y?’ The output of the model is the answer to that question. So in this case either ‘yes, agent X believes Y,’ or ‘no, agent X does not believe Y.’ Such a set-up is often found in experimental tasks, where subjects are asked to reason about the epistemic states of agents in a scenario that they are presented. It might feel more natural to model such cognitive tasks as a search problem, where the question is, for instance, ‘what does agent X believe?’ or ‘what does agent X belief about Y?’ We pose the model in the form of a decision problem, as this is convenient for analyzing its computational complexity. Since the complexity of the decision problem holds as a lower bound for the complexity of such a search problem, considering the decision problem suffices for the purpose at hand.

**Fig. 3:**
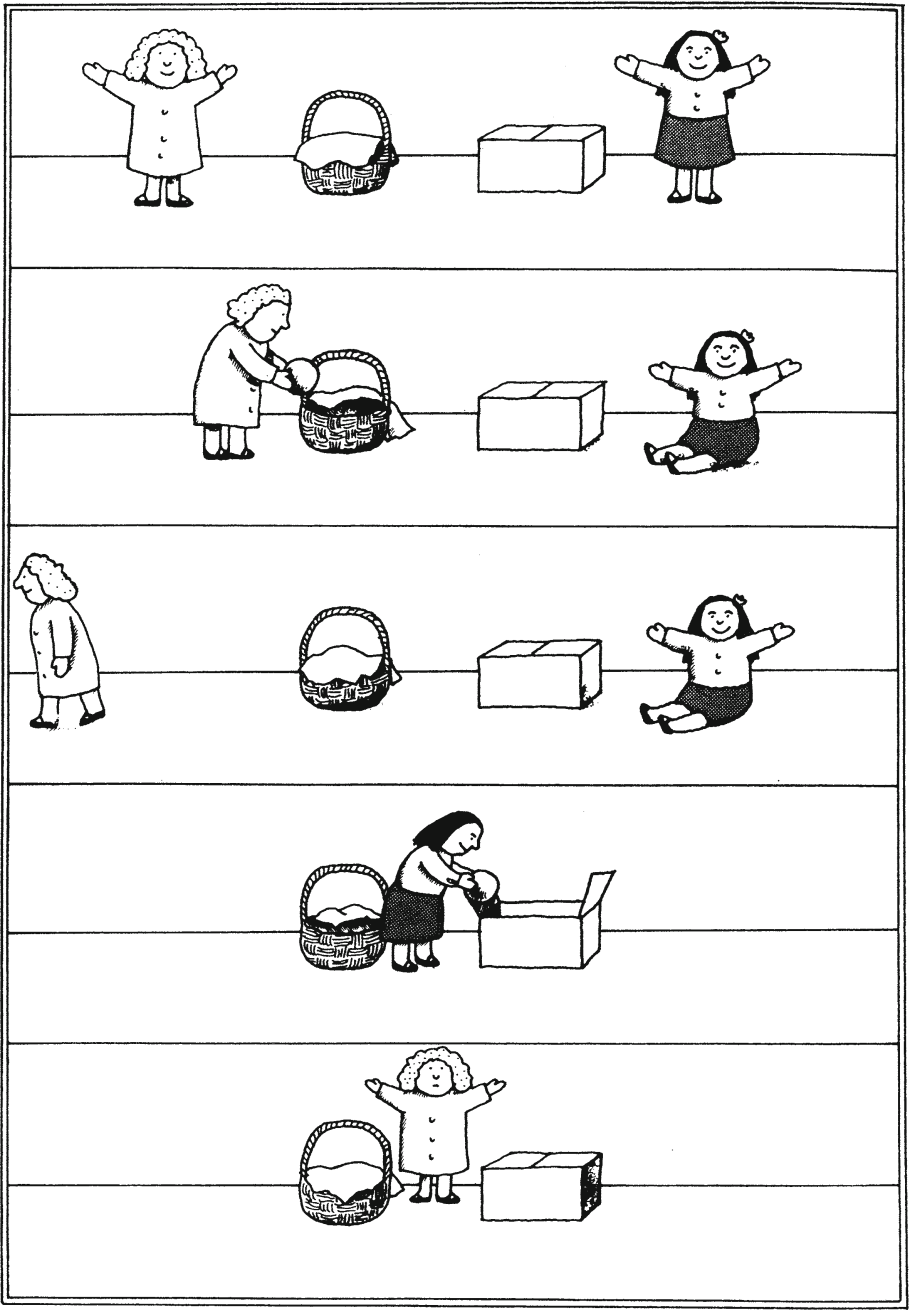
Scenario of the Sally-Anne task from Frith (2001). With kind permission from the artist, Axel Scheffler

Consider the following scenario, which is used in the Sally-Anne task (Baron-Cohen et al. 1985; Wimmer and Perner 1983; see Fig. 3 for an illustration). This is the classical task that is used to test theory of mind, specifically reasoning about false-belief, in young children. Initially, we see Sally and Anne with a box and a basket. Sally has a marble that she puts into the basket. Then Sally leaves and Anne puts the marble into the box. After this, Sally comes back and we are asked where Sally thinks that the marble is. We could model this as an instance of our model, as an input consisting of an initial situation, an event, and a question. In the initial situation both Sally and Anne know that the marble is in the basket. Then there is the event in which Sally leaves and Anne moves the marble into the box. Applying this event to the initial situation leads to a new situation in which the marble is in the box, Anne knows that the marble is in the box, and Sally believes that the marble is in the basket. The question would be: ‘does Sally believe that the marble is in the basket?’ The output of the model would be, ‘yes, Sally believes that the marble is in the basket.’ There are also second-order versions of this false believe task (see Miller 2009). Since we are interested in the effect of higher-order reasoning on the (in)tractability of theory of mind, the second-order version is very relevant to our story. For simplicity we model only the first-order false belief task here to explain how our model works.

**Fig. 4:**
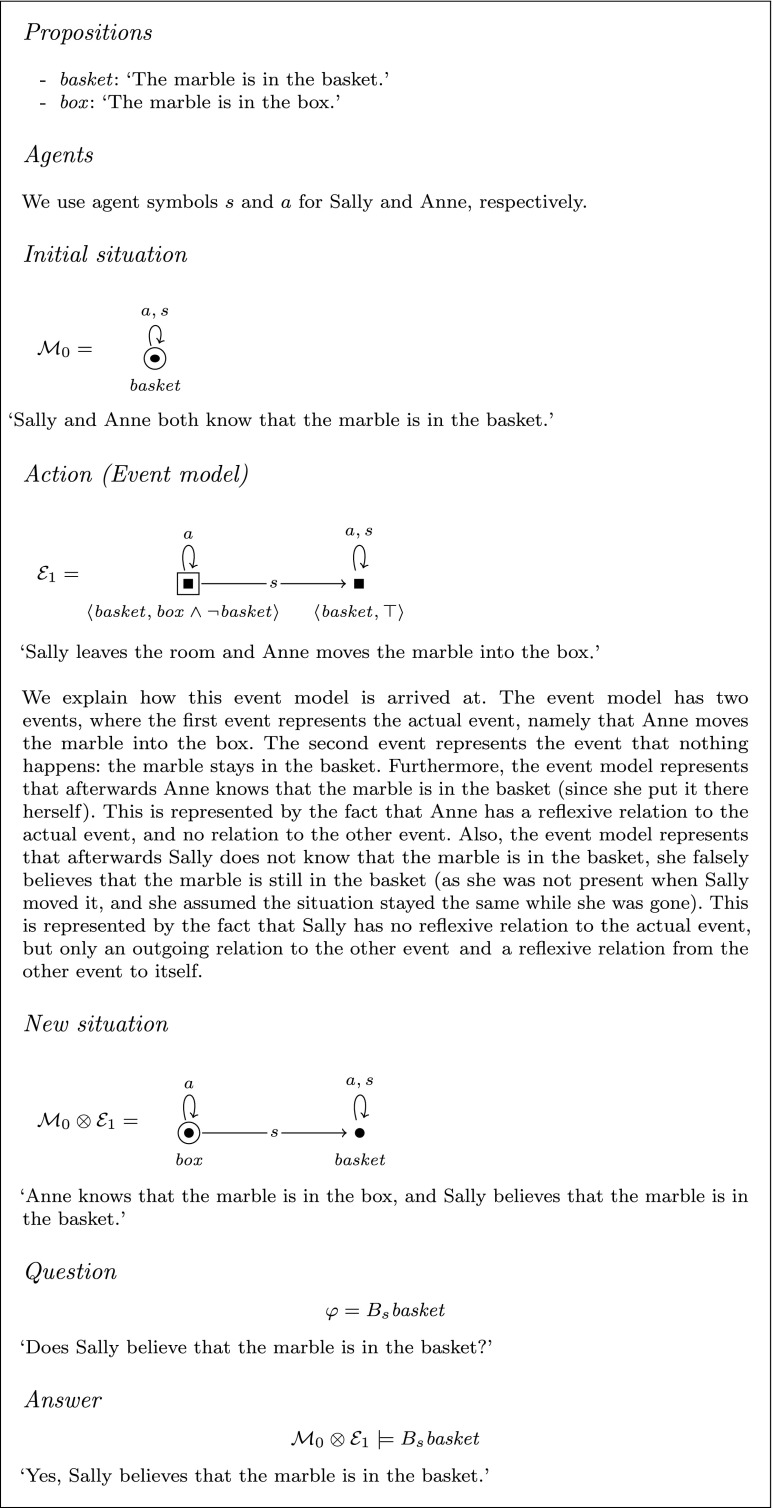
Formalisation of the Sally-Anne task as an instance of the Dynamic Belief Update model

In our model the initial situation is given in the form of a pointed epistemic model $(\mathcal {M}_0, W_d)$, and the actions and events are given as event models $(\mathcal {E}_1,E_1), \ldots , (\mathcal {E}_u, E_u)$. The question is given as a statement $\varphi$ in language $\mathcal {L}_B$, for which we ask whether $\varphi$ is true in the updated model, $(\mathcal {M}_0, W_d) \otimes (\mathcal {E}_1,E_1) \otimes \cdots \otimes (\mathcal {E}_u, E_u)$, i.e., the model that results from updating the initial situation $(\mathcal {M}_0, W_d)$ with event models $(\mathcal {E}_1, E_1)$ to $(\mathcal {E}_u, E_u)$. That is, we ask whether $(\mathcal {M}_0, W_d) \otimes (\mathcal {E}_1, E_1) \otimes \cdots \otimes (\mathcal {E}_u, E_u) \models \varphi$. The full definition of the model is as follows.
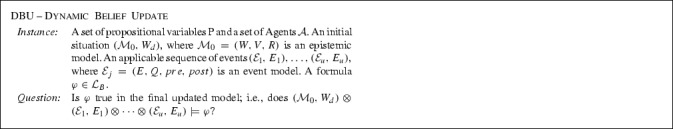


Now we can use this Dynamic Belief Update model to formalize the Sally-Anne task. This formalization is similar to the one presented by Bolander (2014). See Fig. 4 for a graphical presentation of our formalization of the Sally-Anne task.

## Computational Complexity Results

Next, we analyze the computational complexity of the Dynamic Belief Update model that we presented in the previous section. As we explained earlier, our aim is to investigate whether the ‘order’ of higher-order theory of mind reasoning is a source of intractability. We show that, without additional restrictions, the model is $\text { PSPACE}$-hard, and that, possibly counterintuitively from a cognitive science perspective, the ‘order parameter’ is not a source of intractability. We also consider several other parameters of the model to investigate which of them *are* sources of intractability (see Table 1 for an overview of these parameters and Fig. 24 for an overview of the results). We show that only when restricting both the number of event models and the number of events in these event models, the model becomes tractable. We first review some basic notions from both classical and parameterized complexity theory. Readers that are familiar with these notions could skip Sects. 4.1 and 4.2.

### Preliminaries: Classical Complexity Theory

We introduce some basic concepts of classical complexity theory. For a more detailed treatment we refer to textbooks on the topic (e.g., Arora and Barak 2009).

In complexity theory, computational problems are often studied in the form of decision problems. Decision problems represent yes-no questions that are asked about a given input. Let $\Sigma$ be a finite alphabet. A *decision problem* *L* (over $\Sigma$) is a subset of $\Sigma ^*$, where $\Sigma ^*$ is the set of all strings over the alphabet $\Sigma$, i.e., $\Sigma ^* = \bigcup \{\Sigma ^m ; m \in \mathbb {N}\}$. We call $x \in \Sigma ^*$ a *yes-instance* of *L* if and only if $x \in L$, and a *no-instance* if and only if $x \not \in L$. To simplify notation, we usually do not mention the underlying alphabet explicitly.

The concept of efficiently solvable problems is captured by the complexity class P, which denotes the class of all decision problems that can be decided by a polynomial-time algorithm, i.e., an algorithm that runs in time $c_1 \cdot n^{c_2}$ for some constants $c_1$, and $c_2$, where *n* denotes the input size. In order to give evidence that certain problems are intractable (i.e., are not in P), complexity theory offers a theoretical tool that is based on the following complexity class: NP.

The class NP consists of all decision problems for which yes-instances can be verified in polynomial time. Let *L* be a decision problem, and let *x* be an instance of *L*. Then *L* is in class NP if there exists a polynomial-time computable function *f* (a verifier), such that *x* is a yes-instance of *L* if and only if there exists a certificate *u* of polynomial size, such that $f(x,u)=1$. Alternatively, NP can be defined as the class of all decision problems that can be solved by a nondeterministic algorithm that runs in polynomial time. Intuitively, a nondeterministic algorithm is an algorithm that can make guesses during the computation. This algorithm is said to solve the problem if there is at least one sequence of guesses that leads the algorithm to accept. The algorithm is said to run in polynomial time if for all possible sequences of guesses the algorithm terminates in polynomial time.

Another crucial part of this intractability tool is the notion of polynomial-time reductions. Let *L* and $$L'$$ be two decision problems. A *polynomial-time reduction* from *L* to $$L'$$ is a mapping *R* from instances of *L* to instances of $$L'$$ such that: (1) for all instances *x* of *L* it holds that $$x' = R(x) $$ is a yes-instance of $$L'$$ if and only if *x* is a yes-instance of *L*, and (2) *R* is computable in polynomial time.

We can now describe the final notions that we need for the theoretical tool to show intractability: the notions of hardness and completeness for a certain complexity class. Let *L* be a decision problem and K a complexity class. Then (1) *L* is *K-hard* if each problem $L'$ in K is polynomial-time reducible to *L*, and (2) *L* is *K-complete* if *L* is K-hard and in K. Intuitively, problems that are hard for class K belong to the most difficult problems in K.

It follows from the definitions that P $\subseteq$ NP. Furthermore, it is widely believed that P $\ne$ NP (see, e.g., Fortnow 2009; Gasarch 2012). This conjecture implies that NP-hard problems are not polynomial-time solvable. Therefore, showing that a problem is NP-hard gives evidence that this problem is intractable.

Another class that can be used in a similar way to show intractability is the class $\text { PSPACE}$. This class consists of all problems that can be solved by an algorithm that runs in at most polynomial space. Intuitively, this can be seen as an algorithm that uses a polynomial amount of memory. The class $\text { PSPACE}$ is a superset of NP, and a problem that is $\text { PSPACE}$-hard is not polynomial-time solvable, unless $\text { P}= \text { NP}$.

### Preliminaries: Parameterized Complexity Theory

We introduce some basic concepts of parameterized complexity theory. For a more detailed introduction we refer to textbooks on the topic (Downey and Fellows 1999, 2013; Flum and Grohe 2006; Niedermeier 2006).

A parameterized problem is similar to a decision problem. In a parameterized problem each instance is paired to a particular parameter value. Let $\Sigma$ be a finite alphabet. A *parameterized problem* *L* (over $\Sigma$) is a subset of ${\Sigma ^*} \times \mathbb {N}$. For an *instance* (*x*, *k*), we call *x* the *main part* and *k* the *parameter*.

The complexity class FPT, which stands for fixed-parameter tractable, is the direct analogue of the class P in classical complexity. Problems in this class are considered efficiently solvable because the non-polynomial-time complexity inherent in the problem is confined to the parameter and in effect the problem is efficiently solvable even for large input sizes, provided that the value of the parameter is relatively small.

Let $\Sigma$ be a finite alphabet. An algorithm $\mathsf {A}$ with input $(x,k) \in \Sigma \times \mathbb {N}$ runs in *fpt-time* if there exists a computable function *f* and a polynomial *p* such that for all $(x, k) \in \Sigma \times \mathbb {N}$, the running time of $\mathsf {A}$ on (*x*, *k*) is at most $f(k) \cdot p(|x|)$. Algorithms that run in fpt-time are called *fpt-algorithms*. A parameterized problem *L* is *fixed-parameter tractable* if there is an fpt-algorithm that decides *L*. FPT denotes the class of all fixed-parameter tractable problems.

Similarly to classical complexity, parameterized complexity also offers a hardness framework to give evidence that (parameterized) problems are not fixed-parameter tractable. The following notion of reductions plays an important role in this framework.

Let *L* and $L'$ be two parameterized problems. An *fpt-reduction* from *L* to $L'$ is a mapping *R* from instances of *L* to instances of $L'$ for which there is a computable function $g: \mathbb {N}\rightarrow \mathbb {N}$ such that: (1) for all instances (*x*, *k*) of *L* it holds that $(x',k')=R(x,k)$ is a yes-instance of $L'$ if and only if (*x*, *k*) is a yes-instance of *L*, (2) *R* is computable in fpt-time, and (3) $k' \le g(k)$.

Another important part of the hardness framework is the parameterized intractability class W[1]. To characterize this class, we consider the following parameterized problem. 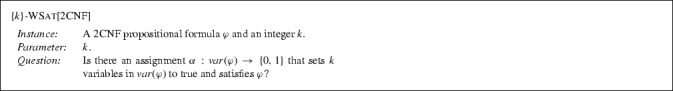


The class W[1] consists of all parameterized problems that can be fpt-reduced to $\{k\}$-WSat[2CNF]. A parameterized problem is hard for W[1] if all problems in W[1] can be fpt-reduced to it. It is widely believed that W[1]-hard problems are not fixed-parameter tractable (see Downey and Fellows 2013).

Another parameterized intractability class, which can be used in a similar way, is the class para-NP. The class para-NP consists of all parameterized problems that can be solved by a nondeterministic algorithm that runs in fpt-time.

W[1] is a subset of para-NP. This implies that para-NP-hard problems are not fixed-parameter tractable, unless W[1] = FPT. In fact, the conjecture P $\ne$ NP already implies that para-NP-hard problems are not fixed-parameter tractable (Flum and Grohe 2006, Theorem 2.14). To show para-NP-hardness, it suffices to show that DBU is NP-hard for a constant value of the parameters (Flum and Grohe 2003).

### PSPACE-Completeness

We show that Dynamic Belief Update (DBU) is PSPACE-complete. Our proof holds for the general case (without any specific restrictions on the relations), and furthermore it holds when the problem is restricted to single-pointed S5 or KD45 models. Since DBU is a special case of DEL model checking, this hardness result also holds for the more general case of DEL model checking that is usually considered in the literature.

Aucher and Schwarzentruber (2013) and van Eijck and Schwarzentruber (2014) showed $\text { PSPACE}$-completeness for DEL model checking in the unrestricted case. Since their proofs depend on the use of multi-pointed models, their results do not hold for the case restricted to single-pointed S5 models. Bolander et al. (2015) considered a special case of DEL model checking that is very similar to the one we consider. They show $\text { PSPACE}$-completeness for the case restricted to single-pointed models, but their proof does not settle whether hardness holds even when the problem is restricted to single-pointed S5 models.

For our reduction, we consider the decision problem True Quantified Boolean Formulas ($\text { TQBF}$), which is the canonical $\text { PSPACE}$-complete problem (Stockmeyer and Meyer 1973). 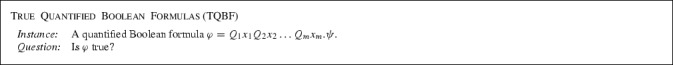


#### Theorem 1

DBU is $\text { PSPACE}$-hard.

#### Proof

To show $\text { PSPACE}$-hardness, we specify a polynomial-time reduction from $\text { TQBF}$ to DBU. Let $\varphi = Q_1 x_1 \ldots Q_m x_m.\psi$ be a quantified Boolean formula with quantifiers $Q_1,\ldots ,Q_m$ and *var*$(\varphi ) = \{x_1, \ldots , x_m\}$, where *var*$(\varphi )$ denotes the set of variables in $\varphi$. We construct a single-pointed epistemic model $(\mathcal {M}, w_0)$, an applicable sequence of single-pointed event models $(\mathcal {E}_1, e_1), \ldots , (\mathcal {E}_m, e_m)$, and a formula $[\varphi ] \in \mathcal {L}_B$, such that $\varphi \in \text { TQBF}{}$ if and only if $(\mathcal {M}, w_0) \otimes (\mathcal {E}_1, e_1) \otimes \cdots \otimes (\mathcal {E}_m, e_m) \models [\varphi ]$.

A main principle of the reduction is that the final updated model $\mathcal {M}^m = (\mathcal {M}, w_0) \otimes (\mathcal {E}_1, e_1) \otimes \cdots \otimes (\mathcal {E}_m, e_m)$ represents exactly all possible truth assignments to *var*$(\varphi )$. We map the variables of $\varphi$, $x_1, \ldots , x_m$, to agents $1, \ldots , m$, and we use these to represent the truth values of the variables $x_1, \ldots , x_m$ under a given assignment. Furthermore, we use a distinguished agent *a* to construct $R_a$-equivalence classes that represent particular truth assignments to *var*$(\varphi )$, and we call these *groups of worlds*. In a group that represents assignment $\alpha$, world *w* represents that $x_i$ is true under $\alpha$ if there is an $R_i$-edge from *w* to a world $w'$ where propositional variable *y* (a new variable, that we introduce) is true. If there is no such world in the group, this represents that $x_i$ is false under $\alpha$.

The other general principle of the reduction is that we use the modal operator *B* and its dual $\hat{B} : = \lnot B \lnot$ to simulate the behavior of the quantifiers in $\varphi$. To define $[\varphi ]$, we first define the following polynomial-time computable mappings. For $1 \le i \le m$, let:

$$\begin{aligned}{}[Q_i] = \left\{ \begin{array}{ll} B_i &{}\quad \hbox { if }Q_i = \forall ; \\ \hat{B_i} &{}\quad \hbox { if } Q_i = \exists . \\ \end{array} \right. \end{aligned}$$

We define $[\psi ]$ to be the adaptation of formula $\psi$ where every occurrence of $x_i$ in $\psi$ is replaced by $\hat{ B_a} \hat{B_i}y$. Now we let $[\varphi ] = [Q_1] \ldots [Q_m] [\psi ]$. So, for example, $[\forall x_1 \exists x_2. x_1 \vee x_2] = B_1 \hat{B_2} ( \hat{B_a} \hat{B_1} y \vee \hat{B_a} \hat{B_2} y)$.

**Fig. 5:**
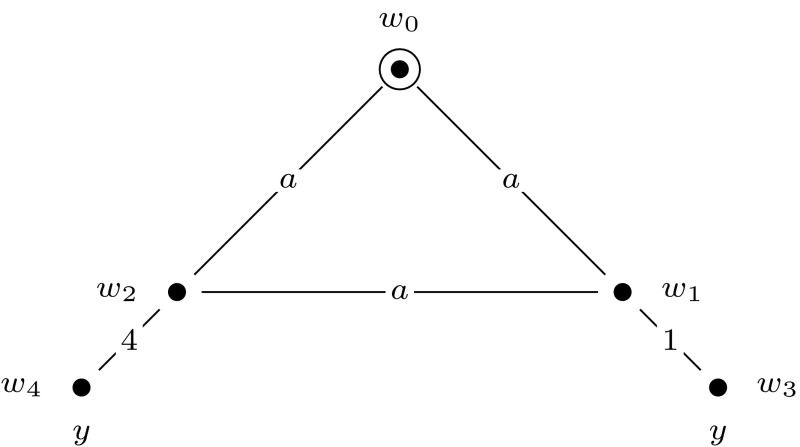
The group of worlds $M = \{w_0, w_1, w_2\}$ that represents assignment $\alpha = \{x_1 \mapsto \text {T}, x_2 \mapsto \text {F}, x_3 \mapsto \text {F}, x_4 \mapsto \text {T}\}$, in the proof of Theorem 1

In Fig. 5 we give an example of such a group of worlds that represents assignment $\alpha = \{x_1 \mapsto \text {T}, x_2 \mapsto \text {F}, x_3 \mapsto \text {F}, x_4 \mapsto \text {T}\}$. Each world has a reflexive relation to itself for every agent, which we omit from our illustrations for the sake of presentation. More generally, in all our drawings we replace each relation $R_a$ with some $R_a'$ whose transitive reflexive closure is equal to $R_a$.

**Fig. 6:**
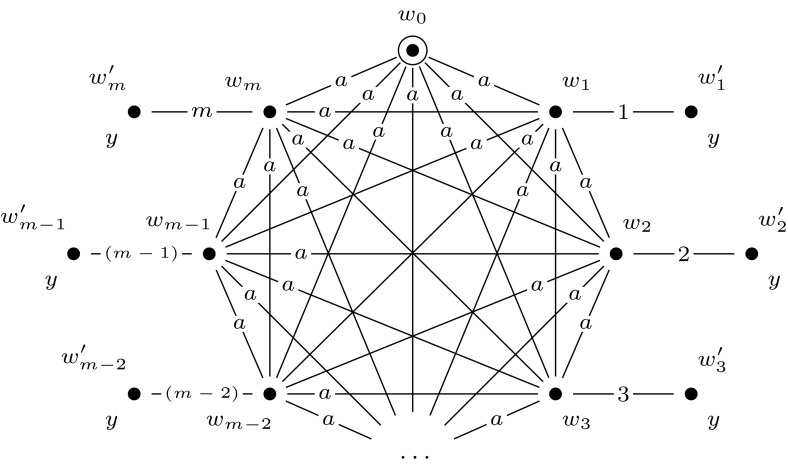
The epistemic model $(\mathcal {M},w_0)$, used in the proofs of Theorem 1, Proposition 2, and Proposition 5

Now, we construct an initial model, $(\mathcal {M},w_{0})$, that represents the truth assignment to *var*$(\varphi )$ that maps every variable of $\varphi$ to true. Model $(\mathcal {M},w_0)$ is a single-pointed epistemic model with agents $a, 1, \ldots , m$ and propositional variable *y*. It consists of worlds $w_0, w_1, \ldots , w_m, w_1', \ldots , w_m'$, of which worlds $w_0, w_1, \ldots , w_m$ are an $R_a$-equivalence class: a group of worlds. See Fig. 6 for an illustration. Again, each world has a reflexive loop for every agent, which we leave out for the sake of presentation. This will be the case for all drawings that we present.

**Fig. 7:**
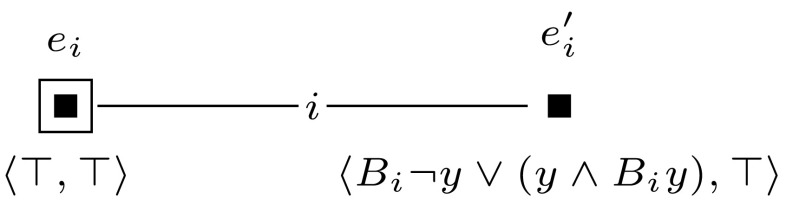
The event model $(\mathcal {E}_i,e_i)$, used in the proof of Proposition 1

Then we construct a sequence of applicable single-pointed event models $(\mathcal {E}_1, e_1), \ldots , (\mathcal {E}_m, e_m)$. For each $1 \le i \le m$, we define $(\mathcal {E}_i, e_i)$ as shown in Fig. 7. Event models $(\mathcal {E}_1, e_1), \ldots , (\mathcal {E}_m, e_m)$ are used to extend the initial model into model $(\mathcal {M}, w_0) \otimes (\mathcal {E}_1, e_1) \otimes \cdots \otimes (\mathcal {E}_m, e_m)$, which represents all possible truth assignments to the variables of $\varphi$. Each event model $(\mathcal {E}_i, e_i)$ copies the entire previous model, and in addition, for each group of worlds in the previous model it adds a slightly adapted group of worlds. This happens in such a way that the assignment that the adapted group of worlds represents, differs from the assignment that the original group of worlds represents, only in the assignment to the variable $x_i$, setting it to false in the adapted group. The preconditions of event model $(\mathcal {E}_i, e_i)$ work as follows. When updating some previous model—either $(\mathcal {M},w_0)$ or some $(\mathcal {M},w_0) \otimes (\mathcal {E}_1, e_1) \otimes \cdots \otimes (\mathcal {E}_{i-1}, e_{i-1})$—with event $(\mathcal {E}_i, e_i)$, event $e_i$ copies the entire previous model. Event $e_i'$ copies an adaptation of each group of worlds by leaving out each pair of worlds $w, w'$, with $wR_iw'$ and $w \ne w'$, that represents variable *i*. It does this by copying only those worlds *w* from which there is no $R_i$-edge to a world in which propositional variable *y* is true, i.e., by copying worlds in which $B_i \lnot y$ is true. In addition, event $e_i'$ also copies worlds $w'$ in which *y* is true and from which there is no $R_i$-edge to a world in which propositional variable *y* is false, i.e., by copying worlds in which $y \wedge B_i y$ is true.

Now, how this reduction works is that in the final updated model $\mathcal {M}^m = (\mathcal {M}, w_0) \otimes (\mathcal {E}_1, e_1) \otimes \cdots \otimes (\mathcal {E}_m, e_m)$ one can, as it were, move from the designated world and visit different groups by taking a path that alternates between different $R_i$-steps, where $1 \le i \le m$. The group that is reachable directly from the designated world represents an assignment where all variables in $\psi$ are set to true. Furthermore, one can walk different paths through the model starting from the designated world, which will bring you to different groups of worlds. Given a group of worlds that represents a particular assignment $\alpha$, taking a (non-reflexive) $R_i$-step will lead to a group of worlds that represents some $\alpha '$ that differs from $\alpha$ only in the value it gives to $x_i$.

**Fig. 8:**
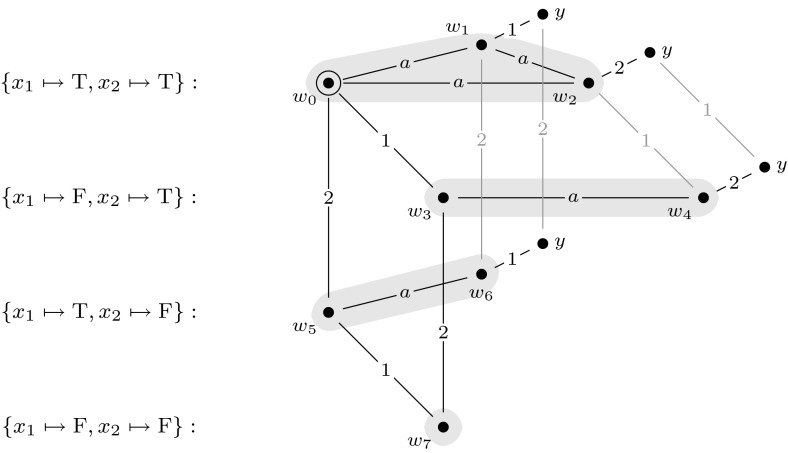
Example for the reduction in the proof of Theorem 1: a final updated model for a quantified Boolean formula with variables $x_1$ and $x_2$. In this model there are four groups of worlds: $\{w_0,w_1,w_2\}$, $\{w_3, w_4\}$, $\{w_5, w_6\}$ and $\{w_7\}$, representing respectively assignments $\{x_1 \mapsto \text {T}, x_2 \mapsto \text {T}\}, \{x_1 \mapsto \text {F}, x_2 \mapsto \text {T}\}, \{x_1 \mapsto \text {T}, x_2 \mapsto \text {F}\}$, and $\{x_1 \mapsto \text {F}, x_2 \mapsto \text {F}\}$

By the way we defined $[\varphi ]$, we can now simulate the behavior of the quantifiers in $\varphi$ and verify whether $\varphi$ is a true quantified Boolean formula by doing model checking for $\mathcal {M}^m$. To illustrate this, we give an example. Figure 8 shows the final updated model for a quantified Boolean formula with variables $x_1$ and $x_2$. In this model there are four groups of worlds: $\{w_0,w_1,w_2\}$, $\{w_3, w_4\}$, $\{w_5, w_6\}$, and $\{w_7\}$, representing respectively assignments $\{x_1 \mapsto \text {T}, x_2 \mapsto \text {T}\}, \{x_1 \mapsto \text {F}, x_2 \mapsto \text {T}\}, \{x_1 \mapsto \text {T}, x_2 \mapsto \text {F}\}$, and $\{x_1 \mapsto \text {F}, x_2 \mapsto \text {F}\}$. Note that for each Boolean formula $\varphi$ with $| {\textit{var}}(\varphi ) |= m$, model $(\mathcal {M}^m, w_0)$ is identical, irrespective of the structure of $\varphi$. The only difference in constructing the reduction is in the construction of $[\varphi ]$.

Then, verifying whether, for instance, formula $\exists x_1 \forall x_2. x_1 \vee x_2$ is true can be done by checking whether $\mathcal {M}^m \models \hat{B}_1 B_2 ( \hat{B}_a \hat{B}_1 y \vee \hat{B}_a \hat{B}_2 y)$, which is indeed the case. This is because the construction of $\mathcal {M}^m$ works in such a way that formula $\exists x_1 \forall x_2. x_1 \vee x_2$ is true if and only if from $w_0$ one can take an $R_1$-step to a group of worlds *M* from where all groups of worlds $M'$ that are reachable by an $R_2$-step represent an assignment that makes $x_{1} \vee x_{2}$ true. In the same manner, verifying whether $\forall x_1 \forall x_2. x_1 \vee x_2$ is true can be done by checking whether $B_1 B_2 (\hat{B}_a \hat{B}_1 y \vee \hat{B}_a \hat{B}_2 y)$ is true in $\mathcal {M}^m$, which is not the case.

We say that group of worlds *M* in model $\mathcal {M}^m$ *agrees* with $\alpha$ if *M* represents some assignment $\beta$ to *var*$(\varphi )$ and $\alpha \subseteq \beta$. We say that *w* is the *base* world of *M* if *w* is the unique world in *M* such that there is no edge from *w* to a world where *y* is true. For example, in Fig. 8, worlds $w_0, w_3,w_5,w_7$ are the base worlds.

We show that $\varphi \in$ $\text { TQBF}$ if and only if $(\mathcal {M}, w_0) \otimes (\mathcal {E}_1, e_1) \otimes \cdots \otimes (\mathcal {E}_m, e_m) \models [\varphi ]$. We do so by proving, with downwards induction, that for all $1 \le i \le m+1$ the following claim holds.

*Claim:* Let $\alpha$ be any truth assignment to the variables $x_1,\ldots ,x_{i-1}$. Let *M* be any group of worlds in model $\mathcal {M}^m=(\mathcal {M}, w_0) \otimes (\mathcal {E}_1, e_1) \otimes \cdots \otimes (\mathcal {E}_m, e_m)$ that represents some assignment $\beta$ to the variables $x_1,\ldots ,x_{m}$, with $\alpha \subseteq \beta$. Let *w* be the base world of *M*. Then $Q_i x_i \ldots Q_m x_m. \psi$ is true under $\alpha$ if and only if $[Q_i]\ldots [Q_m] [\psi ]$ is true in *w*. In the case for $i=m+1$, $Q_i x_i \ldots Q_m x_m. \psi$ refers to formula $\psi$ and $[Q_i]\ldots [Q_m] [\psi ]$ to $[\psi ]$.

We start by showing that the claim holds for $i = m+1$. Let $\alpha$ be any assignment to the variables $x_1, \ldots ,x_m$, and let *w* be the base world of a group of worlds *M* in $\mathcal {M}^m$ that represents assignment $\beta$, with $\alpha \subseteq \beta$. In this case *M* not only agrees with $\alpha$, but in fact $\alpha = \beta$. Since *M* represents assignment $\beta$, then, by construction of $\mathcal {M}^m$, for each variable $x_j$ of $\varphi$ that is true under $\beta$, there is an $R_a$-edge from *w* to a world from which there is an $R_j$-edge to a world where *y* is true, meaning that $(\mathcal {M}^m, w) \models \hat{B}_a \hat{B}_j y$. Similarly, for each variable $x_k$ of $\varphi$ that is false under $\beta$, we have that $(\mathcal {M}^m, w) \not \models \hat{B}_a \hat{B}_{k} y$. Then, by construction of $[\psi ]$, we know that $\psi$ is true under $\alpha$ if and only if $[\psi ]$ is true in *w*. Therefore, the claim holds for $i = m+1$.

Now assume that the claim holds for $i = j + 1$, for an arbitrary $j\ge 1$. We show that then the claim also holds for $i = j$. Let $\alpha$ be any assignment to the variables $x_1, \ldots , x_{j-1}$, and let *w* be the base world of a group *M* that agrees with $\alpha$. We show that the formula $Q_j \ldots Q_m.\psi$ is true under $\alpha$ if and only if $[Q_j] \ldots [Q_m][\psi ]$ is true in *w*.

First, assume that $Q_j \ldots Q_m.\psi$ is true under $\alpha$. Consider the case where $Q_j = \forall$. (The case where $Q_j = \exists$ is analogous.) Then under both assignments $\alpha _1, \alpha _2 \supseteq \alpha$ to the variables $x_1, \ldots , x_{j-1}, x_j$, formula $Q_{j+1} \ldots Q_m.\psi$ is true. Let $\alpha ' \supseteq \alpha$ be any one of these two assignments. Let $M'$ be any group of worlds that agrees with $\alpha '$, and let $w'$ be the base world of $M'$. Now, since $Q_{j+1} \ldots Q_m.\psi$ is true under $\alpha '$ and by the induction hypothesis the claim holds for $j +1$, we know that $[Q_{j+1}] \ldots [Q_m][\psi ]$ is true in $w'$.

Since $Q_j = \forall$, we know that $[Q_j] = B_j$. So if we can show that $[Q_{j+1}] \ldots [Q_m][\psi ]$ is true in all worlds that are $R_j$-reachable from *w*, then we know that $[Q_{j}] \ldots [Q_m][\psi ]$ is true in *w*. By construction of $\mathcal {M}^m$, all equivalence classes for $R_1, \ldots , R_m$ are of size two because this is the case in model $(\mathcal {M}, w_0)$, and this property is kept intact by the event models. So the worlds that are $R_j$-reachable from *w* are *w* itself and some world $w^* \ne w$. Furthermore, by construction of $\mathcal {M}^m$, for any base world *w* of some group *M* there is exactly one world $w^* \ne w$ that is $R_j$-reachable from *w*, and $w^*$ is the base world of some group $M^* \ne M$. The assignment that $M^*$ represents differs from the assignment that *M* represents only in the assignment to the variable $x_i$.

Now assume without loss of generality that group *M* agrees with assignment $\alpha _1$, then group $M^*$ agrees with $\alpha _2$. Therefore, by the induction hypothesis, $[Q_{j+1}] \ldots [Q_m][\psi ]$ is true in both *w* and $w^*$, and thus in all worlds that are $R_j$-reachable from *w*. Hence, $[Q_{j}] \ldots [Q_m][\psi ]$ is true in *w*.

Next, assume that $Q_j \ldots Q_m.\psi$ is not true under $\alpha$. Consider the case where $Q_j = \forall$. (The case where $Q_j = \exists$ is analogous.) Then there is some assignment $\alpha ' \supseteq \alpha$ to the variables $x_1, \ldots , x_j$, such that $Q_{j+1} \ldots Q_m.\psi$ is not true under $\alpha '$. Let $M'$ be the group of worlds that agrees with $\alpha '$, and let $w'$ be the base world of $M'$. Now, by the induction hypothesis, we know that $[Q_{j+1}] \ldots [Q_m][\psi ]$ is not true in $w'$. Furthermore, since $Q_j = \forall$, we know that $[Q_j] = B_j$. So if we can show that there is some world that is $R_j$-reachable from *w* in which $[Q_{j+1}] \ldots [Q_m][\psi ]$ is not true, then we know that $[Q_{j}] \ldots [Q_m][\psi ]$ is not true in *w*. We know that $w'$ is $R_j$-reachable from *w* by construction of $\mathcal {M}^m$ and the fact that the assignments that *M* and $M'$ represent differ only by variable $x_j$. Hence $[Q_{j}] \ldots [Q_m][\psi ]$ is not true in *w*.

Therefore, the claim holds for the case that $i = j$. Now, by induction, the claim holds for the case that $i = 1$, and hence it follows that $\varphi \in \text { TQBF}{}$ if and only if $(\mathcal {M}, w_0) \otimes (\mathcal {E}_1, e_1) \otimes \cdots \otimes (\mathcal {E}_m, e_m) \models [\varphi ]$. Since this reduction runs in polynomial time, we conclude that DBU is $\text { PSPACE}$-hard. $\square$

#### Theorem 2

DBU is $\text { PSPACE}$-complete.

#### Proof

In order to show PSPACE-membership for DBU, we can modify the polynomial-space algorithm given by Aucher and Schwarzentruber (2013). Their algorithm works for the problem of checking whether a given single-pointed epistemic model makes a given DEL-formula true, where that formula may contain event models that can be multi-pointed but have no postconditions. To make their algorithm work for our formalism, it needs to be able to deal with multi-pointed epistemic models and event models with postconditions. In order to make their algorithm work for multi-pointed epistemic models, we can call the algorithm several times: once for each of the designated worlds. Furthermore, a modification is needed to deal with postconditions. Their algorithm checks the truth of a formula by inductively calling itself for subformulas. In order to deal with postconditions, only the case where the formula is a propositional variable needs to be modified. Instead of just checking whether the variable is true in the initial model, one needs to take into account whether this variable has been set to true or false by the postconditions. $\square$

### Parameterized Complexity Results

Next, we provide a parameterized complexity analysis of Dynamic Belief Update (DBU).

#### Parameters for DBU

We consider the following parameters for DBU. For each subset $\kappa \subseteq \{ a,c,e,f,o,p,u \}$ we consider the parameterized variant $\kappa \text { -}$ DBUof DBU, where the parameter is the sum of the values for the elements of $\kappa$ as specified in Table 1. For instance, the problem $\{a,p\}\text {-DBU}$ is parameterized by the number of agents plus the number of propositional variables. Even though technically speaking there is only one parameter, we will refer to each of the elements of $\kappa$ as parameters. For every set $\kappa$ of parameters it holds that if $\kappa \text { -}$ DBU  is fixed-parameter tractable, then for every $\kappa ' \supseteq \kappa$, $\kappa '\text { -}$ DBU  is also fixed-parameter tractable.

For the modal depth of a formula we count the maximum number of nested occurrences of modal operator *B*. Formally, we define the modal depth $d(\varphi )$ of a formula $\varphi$ (in $\mathcal {L}_B$) recursively as follows.

$$\begin{aligned} d(\varphi ) = \left\{ \begin{array}{ll} 0&{}\quad \hbox {if }\varphi = p\in P\hbox { is a propositional variable};\\ \max \{d(\varphi _1), d(\varphi _2) \}&{}\quad \hbox {if }\varphi = \varphi _1 \wedge \varphi _2;\\ d(\varphi _1) &{}\quad \hbox {if }\varphi = \lnot \varphi _1;\\ 1 + d(\varphi _1) &{}\quad \hbox {if } \varphi = B_a \varphi _1 ,\hbox { for some agent }a.\\ \end{array} \right. \end{aligned}$$

For the size of a formula we count the number of occurrences of propositional variables and logical connectives. Formally, we define the size $s(\varphi )$ of a formula $\varphi$ (in $\mathcal {L}_B$) recursively as follows.

$$\begin{aligned} s(\varphi ) = \left\{ \begin{array}{ll} 1&{}\quad \hbox { if }\varphi = p \in P\hbox { is a propositional variable};\\ 1 + s(\varphi _1) + s(\varphi _2) &{}\quad \hbox { if }\varphi = \varphi _1 \wedge \varphi _2;\\ 1 + s(\varphi _1) &{} \quad \hbox { if }\varphi = \lnot \varphi _1;\\ 1 + s(\varphi _1) &{} \quad \hbox { if } \varphi = B_a \varphi _1, \hbox { for some agent } a.\\ \end{array} \right. \end{aligned}$$

**Table 1:** Overview of the different parameters for DBU

Param.	Description
*a*	Number of agents
*c*	Maximum size of the preconditions
*e*	Maximum number of events in the event models
*f*	Size of the formula
*o*	Modal depth of the formula, i.e., the order parameter
*p*	Number of propositional variables in *P*
*u*	Number of event models, i.e., the number of updates

#### Fixed-Parameter Intractability Results

In the following, we show fixed-parameter intractability for several parameterized versions of DBU. We will use the parameterized complexity classes W[1] and para-NP to show intractability; i.e., we will show hardness for these classes. Note that we could additionally use the class para-PSPACE (Flum and Grohe 2003) to give stronger intractability results. For instance, the proof of Theorem 1 already shows that $\{p\}$-DBU is para-PSPACE hard since the reduction in this proof uses a constant number of propositional variables. However, since in this paper we are mainly interested in the border between fixed-parameter tractability and intractability, we will not focus on the subtle differences in the degree of intractability and restrict ourselves to showing W[1]-hardness and para-NP-hardness. This is also the reason why we will not show membership for any of the (parameterized) intractability classes; showing hardness suffices to indicate intractability. For the following proofs we use the well-known satisfiability problem Sat for propositional formulas, which is the canonical NP-complete problem (Cook 1971; Levin 1973). We use the fact that hardness for Sat holds even when restricted to propositional formulas that are in 3CNF (Cook 1971).

##### Proposition 1

$\{a,c,e,f,o\}$-DBU is para-NP-hard.

##### Proof

To show para-NP-hardness, we specify a polynomial-time reduction from Sat to DBU, where parameters *a*, *c*, *e*, *f*, and *o* have constant values. Let $\varphi$ be a propositional formula with *var*$(\varphi ) = \{x_1, \ldots , x_m\}$. Without loss of generality we assume that $\varphi$ is a 3CNF formula with clauses $c_1$ to $c_l$. We introduce a propositional variable $z_{m+1}$ and construct a single-pointed epistemic model $(\mathcal {M}, w_0)$ and an applicable sequence of single-pointed event models $(\mathcal {E}_1, e_1), \ldots , (\mathcal {E}_{m+l}, e_{m+l})$, such that $\varphi \in$ Sat if and only if $(\mathcal {M}, w_0) \otimes (\mathcal {E}_1, e_1) \otimes \cdots \otimes (\mathcal {E}_{m+l}, e_{m+l}) \models z_{m+1}$.

The general idea behind this reduction is that we use the individual worlds in model $\mathcal {M}^{m} = (\mathcal {M}, w_0) \otimes (\mathcal {E}_1, e_1) \otimes \cdots \otimes (\mathcal {E}_m, e_{m})$ to list all possible assignments to *var*$(\varphi )$. We do this by introducing a propositional variable $z_i$ for each variable $x_i$ in $\varphi$ and by constructing $\mathcal {M}^{m}$ in such a way that the valuation $V^m$ over these propositional variables, in a given world *w*, represents an assignment $\alpha$ over $var(\varphi )$, i.e. $\alpha (x_i) = V^m(w,z_i)$. So each world in model $\mathcal {M}^m$ represents an assignment $\alpha$ over $var(\varphi )$, and each assignment $\alpha$ over $var(\varphi )$ is represented by a world in model $\mathcal {M}^m$.

**Fig. 9:**
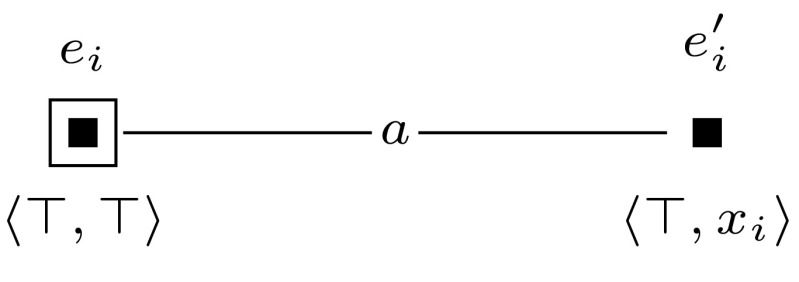
The event model $(\mathcal {E}_i,e_i)$, used in the proof of Proposition 1

We let $(\mathcal {M},w_0)$ be a single-agent, single-pointed epistemic model, consisting of a single world, in which all propositional variables $z_i$ are set to false. Furthermore, we construct a sequence of applicable single-pointed event models $(\mathcal {E}_1, e_1), \ldots , (\mathcal {E}_m, e_m)$. For each $1 \le i \le m$, we define $(\mathcal {E}_i, e_i)$ as shown in Fig. 9. Now, checking whether formula $\varphi$ is satisfiable can be done by checking whether $\varphi$ is true in any of the worlds in $\mathcal {M}^{m}$.

**Fig. 10:**
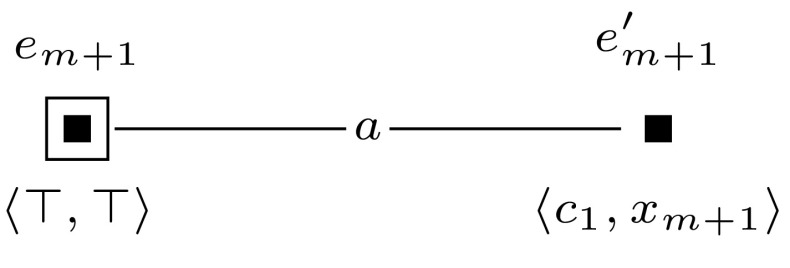
The event model $(\mathcal {E}_{m+1},e_{m+1})$, used in the proof of Proposition 1

**Fig. 11:**
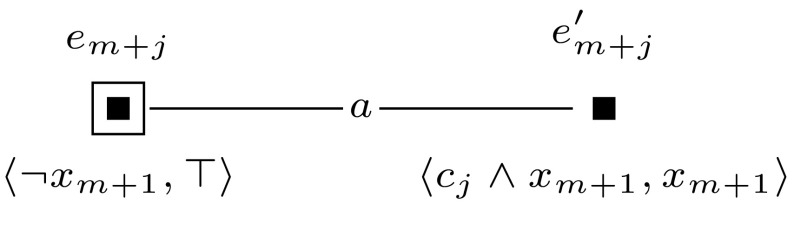
The event model $(\mathcal {E}_{m+j},e_{m+j})$, used in the proof of Proposition 1

Furthermore, to keep the formula that we check in the final updated model  $\mathcal {M}^{m+l} = (\mathcal {M}, w_0) \otimes (\mathcal {E}_1, e_1) \otimes \cdots \otimes (\mathcal {E}_{m+l}, e_{m+l})$ of constant size, we sequentially check the truth of each clause $c_i$ and encode whether the clauses are true with an additional variable $z_{m+1}$. This is done by event models $(\mathcal {E}_{m+1}, e_{m+1})$ to $(\mathcal {E}_{m+l}, e_{m+l})$, which we define for $m+1$ and for each *j*, $m+2 \le j \le m+l$ as shown in Figures 10 and 11.

In the final updated model $\mathcal {M}^{m+l}$, variable $z_{m+1}$ is true in a world *w* if and only if clauses $c_1$ to $c_l$ are true in *w*, i.e., if it makes formula $\varphi$ true. Hence, we can conclude that $\varphi \in$ Sat if and only if $(\mathcal {M}, w_0) \otimes (\mathcal {E}_1, e_1) \otimes \cdots \otimes (\mathcal {E}_{m+l}, e_{m+l}) \models z_{m+1}$. $\square$

##### Proposition 2

$\{c,e,f,o,p\}\text {-DBU}$ is para-NP-hard.

##### Proof

To show para-NP-hardness, we specify a polynomial-time reduction *R* from Sat to DBU, where parameters *c*, *e*, *f*, *o*, and *p* have constant values. Let $\varphi$ be a propositional formula with *var*$(\varphi ) = \{x_1, \ldots , x_m\}$. Without loss of generality we assume that $\varphi$ is a 3CNF formula with clauses $c_1$ to $c_l$. We introduce a propositional variable *z* and agents *b* and *c*, and we construct a single-pointed epistemic model $(\mathcal {M}, w_0)$ and an applicable sequence of single-pointed event models $(\mathcal {E}_1, e_1), \ldots , (\mathcal {E}_{m+l}, e_{m+l})$, such that $\varphi \in$ Sat if and only if $(\mathcal {M}, w_0) \otimes (\mathcal {E}_1, e_1) \otimes \cdots \otimes (\mathcal {E}_{m+l}, e_{m+l}) \models \hat{B}_b \hat{B}_c z$.

**Fig. 12:**
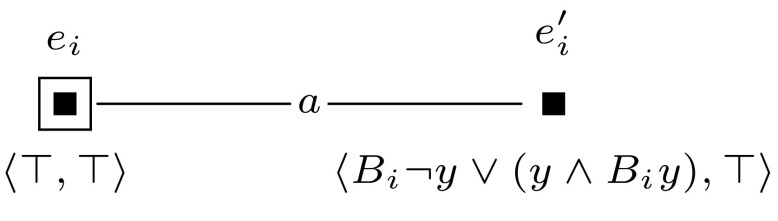
The event model $(\mathcal {E}_i,e_i)$, used in the proof of Proposition 2

The general idea behind this reduction is similar to the reduction in the proof of Theorem 1. Again, we use groups of worlds to represent particular assignments to the variables in $\varphi$. To construct these, we use a single-pointed epistemic model $(\mathcal {M},w_0)$ with agents $a, 1, \ldots , m$ and propositional variable *y*, such as defined in Fig. 6. Furthermore, we use a sequence of applicable single-pointed event models $(\mathcal {E}_1, e_1), \ldots , (\mathcal {E}_m, e_m)$. For each $1 \le i \le m$, we define $(\mathcal {E}_i, e_i)$ as shown in Fig. 12.

**Fig. 13:**
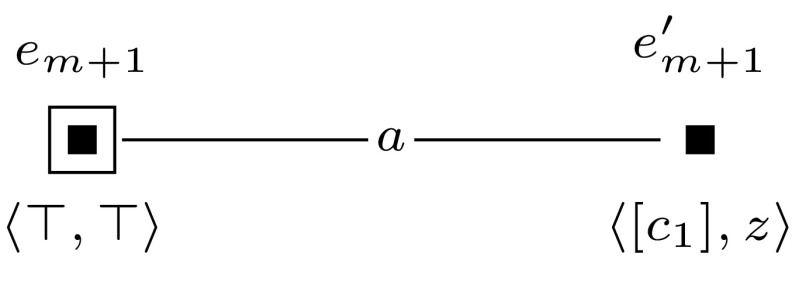
The event model $(\mathcal {E}_{m+1},e_{m+1})$, used in the proof of Proposition 2. Here, $[c_1]$ is the adaptation of clause $c_1$, where every occurrence of $x_i$ in $c_1$ is replaced by $\hat{B}_a\hat{B}_i y$

**Fig. 14:**
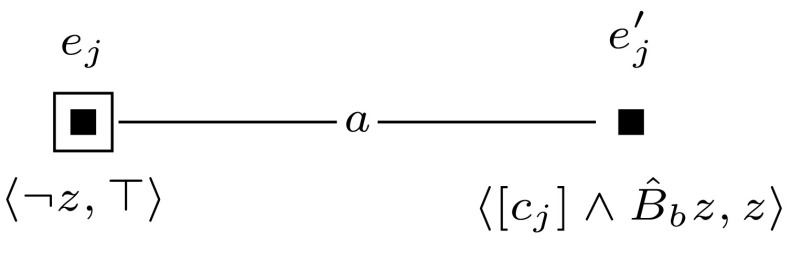
The event model $(\mathcal {E}_j,e_j)$, used in the proof of Proposition 2. Here, $[c_j]$ is the adaptation of clause $c_j$, where every occurrence of $x_i$ in $c_j$ is replaced by $\hat{B}_a\hat{B}_i y$

To keep the formula that we check in the final updated model $\mathcal {M}^{m+l} = (\mathcal {M}, w_0) \otimes (\mathcal {E}_1, e_1) \otimes \cdots \otimes (\mathcal {E}_{m+l}, e_{m+l})$ of constant size, we sequentially check the truth of each clause $c_i$ and encode whether the clauses are true with an additional variable *z*. This is done by the single-pointed event models $\mathcal {E}_{m+1}$ to $\mathcal {E}_{m+l}$. For $m+1$ and for $m+2 \le j \le m+l$, we define event models $(\mathcal {E}_{m+1}, e_{m+1})$ and $(\mathcal {E}_j, e_j)$ as shown in Figures 13 and 14. Event model $e_{j}$ (corresponding to clause *j*) marks each group of worlds that represents an assignment that satisfies clauses 1 to *j*. It makes sure that each group of worlds that represents a satisfying assignment for the given formula will have an $R_c$ relation from a world that is $R_b$-reachable from the designated world to a world where propositional variable *z* is true.

In the final updated model, there will be such a marked group if and only if all clauses are satisfiable (and thus if and only if the whole formula is satisfiable). Hence, we can conclude that $\varphi \in$ Sat if and only if $(\mathcal {M}, w_0) \otimes (\mathcal {E}_1, e_1) \otimes \cdots \otimes (\mathcal {E}_{m+l}, e_{m+l}) \models \hat{B}_b \hat{B}_c z$. $\square$

##### Proposition 3

$\{a,e,f,o,p\}\text {-DBU}$ is para-NP-hard.

##### Proof

To show para-NP-hardness, we specify a polynomial-time reduction *R* from Sat to DBU, where parameters *a*, *e*, *f*, *o*, and *p* have constant values. Let $\varphi$ be a propositional formula with *var*$(\varphi ) = \{x_1, \ldots , x_m\}$. Without loss of generality we assume that *m* is even. We introduce a propositional variable $z^*$ and agents *b* and *c*, and we construct a single-pointed epistemic model $(\mathcal {M}, w_0)$ and an applicable sequence of single-pointed event models $(\mathcal {E}_1, e_1), \ldots , (\mathcal {E}_{m+1}, e_{m+1})$, such that $\varphi \in$ Sat if and only if $(\mathcal {M}, w_0) \otimes (\mathcal {E}_1, e_1) \otimes \cdots \otimes (\mathcal {E}_{m+1}, e_{m+1}) \models \hat{B}_b \hat{B}_c z^*$.

The reduction is based on the same principle as the one used in the proof of Theorem 1. Again, we use groups of worlds to represent particular assignments to the variables in $\varphi$. To keep the number of agents constant, we use a different construction to represent the variables in $\varphi$. We encode the variables by a string of worlds that are connected by alternating relations $R_a$ and $R_b$. We use the folowing polynomial-time computable mappings. For $1 \le i \le m$, we define $[x_i]$ inductively as follows: $[x_1] = \hat{B}_b$,

$$\begin{aligned} {[}x_{j+1}] = \left\{ \begin{array}{ll} {[}x_j] {\hat{B}}_b &{} \quad \hbox {if }[x_j]\hbox { ends with } \hat{B}_a, \\ {[}x_j] {\hat{B}}_a &{} \quad \hbox {if }[x_j]\hbox { ends with } \hat{B}_b.\\ \end{array} \right. \end{aligned}$$

Then $[\varphi ]$ is the adaptation of formula $\varphi$, where for $2 \le i \le m$, every occurrence of $x_i$ in $\varphi$ is replaced by $\hat{B}_a ([x_i]y \wedge \lnot [x_{i-1}]y)$, and every occurrence of $x_1$ is replaced by $\hat{B}_a[x_1]$. Let *M* be a group of worlds, and let *w* be the unique world in *M* such that there is no world accessible from *w*, in one step, where *y* is true. We say that *M* represents an assignment $\alpha$ to variables $x_1, \ldots , x_m$ if (1) for each $2 \le i \le m$, if $\alpha$ sets $x_i$ to true, then $\hat{B}_a ([x_i]y \wedge \lnot [x_{i-1}]y)$ is true in *w*, and (2) if $\alpha$ sets $x_1$ to true, then $\hat{B}_a[x_1] y$ is true in *w*.

**Fig. 15:**
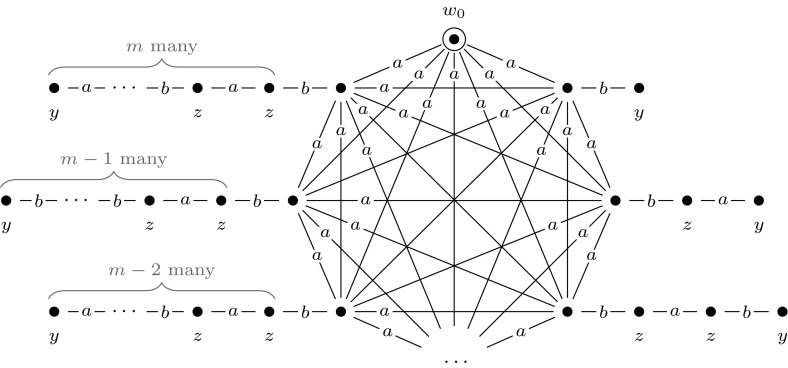
The epistemic model $(\mathcal {M},w_0)$, used in the proofs of Proposition 3 and Proposition 6

**Fig. 16:**
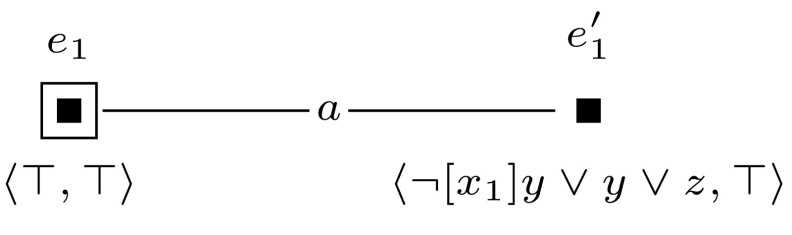
The event model $(\mathcal {E}_1,e_1)$, used in the proof of Proposition 3

**Fig. 17:**
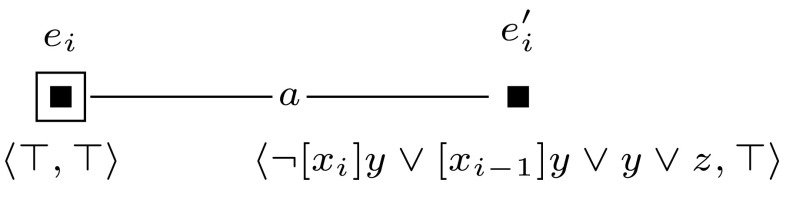
The event model $(\mathcal {E}_i,e_i)$, used in the proof of Proposition 3

To construct this model with groups of worlds we introduce a single-pointed epistemic model $(\mathcal {M},w_0)$ with agents *a* and *b*, and propositional variables *y* and *z*, such as defined in Fig. 15. Furthermore, we use a sequence of applicable single-pointed event models $(\mathcal {E}_1, e_1), \ldots , (\mathcal {E}_m, e_m)$. We define $(\mathcal {E}_1, e_1)$ as shown in Fig. 16, and for each $2 \le i \le m$, we define $(\mathcal {E}_i, e_i)$ as shown in Fig. 17.

**Fig. 18:**
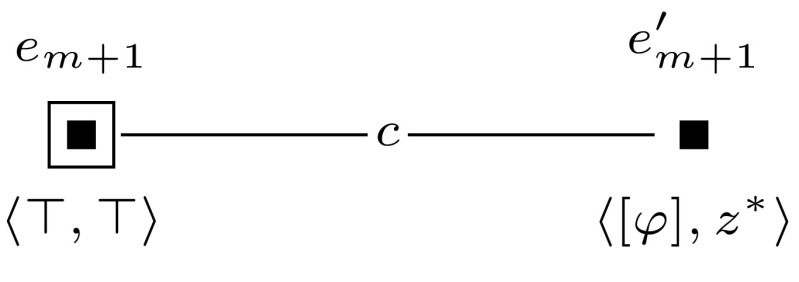
The event model $(\mathcal {E}_{m+1},e_{m+1})$, used in the proof of Proposition 3

Furthermore, we keep the size of the formula constant by encoding the satisfiability of the formula with a single propositional variable. We do this using event model $(\mathcal {E}_{m+1}, e_{m+1})$ with agent *c* and propositional variable $z^*$, as defined in Fig. 18.

Event model $(\mathcal {E}_{m+1}, e_{m+1})$ makes sure that each group of worlds that represents a satisfying assignment for formula $\varphi$ will be marked; namely, each such group will have an $R_c$ relation from a world that is $R_b$-reachable from the designated world to a world where propositional variable $z^*$ is true. Then in the final updated model $\mathcal {M}^{m+1} = (\mathcal {M}, w_0) \otimes (\mathcal {E}_1, e_1) \otimes \cdots \otimes (\mathcal {E}_{m+1}, e_{m+1})$ there will be such a marked group if and only if formula $\varphi$ is satisfiable, i.e.,  $\varphi \in$ Sat if and only if $\mathcal {M}^{m+1} \models \hat{B}_b \hat{B}_c z$. $\square$

We consider the following parameterized problem, that we use in our proof of Proposition 4. This problem is W[1]-complete (Fellows et al. 2009). 
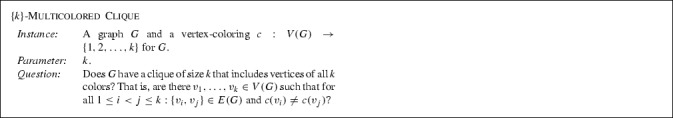

##### Proposition 4

$\{a,c,f,o,u\}$-DBU is W[1]-hard.

##### Proof

To show W[1]-hardness, we specify an fpt-reduction *R* from $\{k\}\text {-{Multicolored Clique}}$ to $\{a,c,f,o,u\}$-DBU. Let (*G*, *c*) be an instance of $\{k\}\text {-{Multicolored Clique}}$, where $G=(N,E)$. We construct a formula $\varphi$, a single-pointed epistemic model $(\mathcal {M}, w_0)$, and an applicable sequence of single-pointed event models $$(\mathcal {E}_1, e_1), \ldots , (\mathcal {E}_{k+\left( {\begin{array}{c}k\\ 2\end{array}}\right) }, e_{k+\left( {\begin{array}{c}k\\ 2\end{array}}\right) })$$ such that $(G,c) \in$ $\{k\}\text {-{Multicolored Clique}}$ if and only if $$(\mathcal {M}, w_0) \otimes (\mathcal {E}_1, e_1) \otimes \cdots \otimes (\mathcal {E}_{k+\left( {\begin{array}{c}k\\ 2\end{array}}\right) }, e_{k+\left( {\begin{array}{c}k\\ 2\end{array}}\right) }) \models \varphi $$.

The general idea behind this reduction is that we use event models $(\mathcal {E}_1, e_1), \ldots , (\mathcal {E}_{k}, e_{k})$ to list all *k*-sized subsets of differently colored vertices of graph *G* in model $\mathcal {M}^k = (\mathcal {M}, w_0) \otimes \cdots \otimes (\mathcal {E}_{k}, e_{k})$. Each world in $\mathcal {M}^k$ represents a particular *k*-colored, *k*-sized subset of vertices in graph *G*. Then with event models $$(\mathcal {E}_{k+1}, e_{k+1}), \ldots , (\mathcal {E}_{k+\left( {\begin{array}{c}k\\ 2\end{array}}\right) }, e_{k+\left( {\begin{array}{c}k\\ 2\end{array}}\right) })$$ we encode the existing edges between these vertices in model $$\mathcal {M}^{k+\left( {\begin{array}{c}k\\ 2\end{array}}\right) } = \mathcal {M}^k \otimes (\mathcal {E}_{k+1}, e_{k+1}) \otimes \cdots \otimes (\mathcal {E}_{k+\left( {\begin{array}{c}k\\ 2\end{array}}\right) }, e_{k+\left( {\begin{array}{c}k\\ 2\end{array}}\right) })$$. Then we can check in $$\mathcal {M}^{k+\left( {\begin{array}{c}k\\ 2\end{array}}\right) }$$ whether there is a world that represents a *k*-colored, *k*-sized subset of vertices that is pairwise fully connected with edges. This is the case if and only if *G* has a *k*-clique with *k* different colors.

**Fig. 19:**
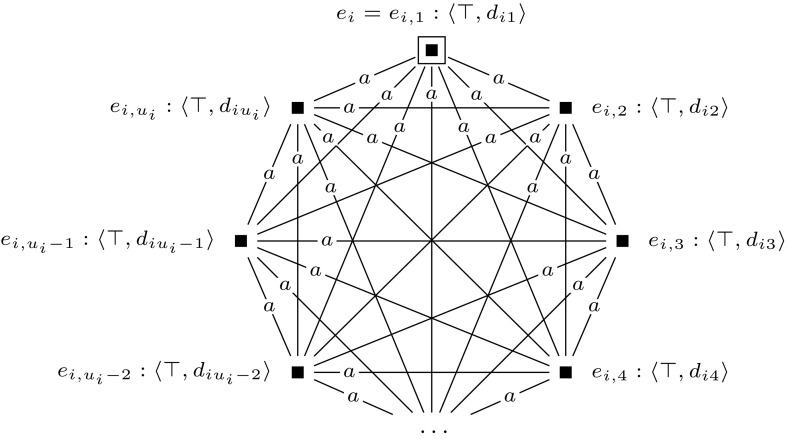
The event model $(\mathcal {E}_i, e_i)$, used in the proof of Proposition 4

For each vertex $v_i \in N$ we introduce propositional variable $x_i$. We let $(\mathcal {M},w_0)$ be a single-agent, single-pointed epistemic model, consisting of a single world, in which all propositional variables are set to false. Furthermore, we construct a sequence of applicable single-pointed event models $(\mathcal {E}_1, e_1), \ldots , (\mathcal {E}_k, e_k)$. Let $X = \{x_1, \ldots , x_{|N|}\}$, we define $D_1 =\{d_{11},\ldots , d_{1u_1}\} = \{x_i \in X ; c(x_i)=1\}{} , \ldots , D_k =\{d_{k1},\ldots , d_{ku_k}\} = \{x_i \in X ; c(x_i)=k\}$. The set $D_j$ contains a propositional variable for each vertex *v* in *N* that has color *j*, and $u_j$ is the number of vertices in *N* that have color *j*. (Note that these $d_{ij}$ are names for propositional variables of the form $x_i$.) For each $1 \le i \le m$, we define $(\mathcal {E}_i, e_i)$ as shown in Fig. 19.

We say that a world *w* represents a *k*-colored, *k*-sized subset of vertices $v_{i_1},\ldots ,v_{i_k}$ in graph *G* if propositional variables $x_{i_1},\ldots ,x_{i_k}$ are true in *w* and all other $x_i$ are false in *w*. Note that all *k*-colored, *k*-sized subsets of vertices in graph *G* are represented by a world in model $\mathcal {M}^k = (\mathcal {M}, w_0) \otimes \cdots \otimes (\mathcal {E}_{k}, e_{k})$, and every world in $\mathcal {M}^k$ represents a *k*-colored, *k*-sized subset of vertices in graph *G*.

Let $$t = \left( {\begin{array}{c}|N|\\ 2\end{array}}\right) $$ and let the set of all possible edges between the vertices in N be $E' = \{\{x'_1, x''_1\},\ldots ,\{x'_{t}, x''_{t}\}\}$. (Note that $x'_\ell$ and $x''_\ell$ are names for propositional variables of the form $x_i$.) We introduce a propositional variable *z*, and a propositional variable $r_{ij}$ for each $1 \le i<j \le k$. We will use propositional variable $r_{ij}$ to encode in some world *w* whether in the subset of vertices that *w* represents there are a vertex *v* with color *i* and a vertex *w* with color *j* that are connected by an edge $\{v,w\}$ in *G*. For each $\{x'_l, x''_l\} \in E'$ we define:

$$\begin{aligned} \psi _l = \left\{ \begin{array}{ll} r_{ij} &{}\quad \hbox {if } \{x'_l, x''_l\} \in E\hbox { and }c(x'_l) = i, c(x''_l) = j, i < j, \\ z &{}\quad \hbox {otherwise. }\\ \end{array} \right. \end{aligned}$$

**Fig. 20:**
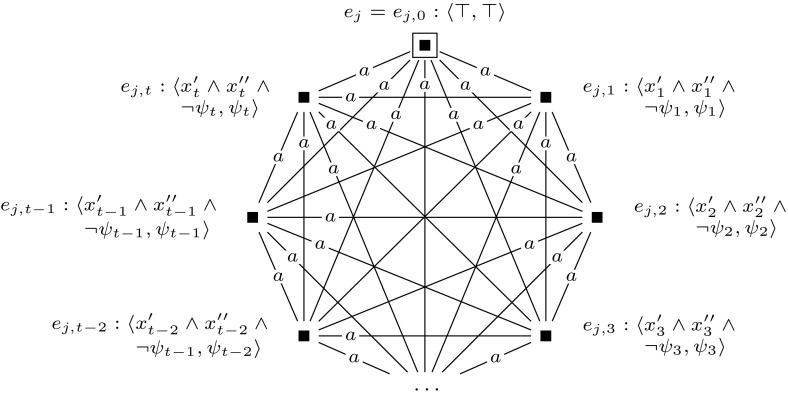
The event model $(\mathcal {E}_j, e_j)$, used in the proof of Proposition 4

Now for each $$k_1 \le j \le k+\left( {\begin{array}{c}k\\ 2\end{array}}\right) $$, we define $(\mathcal {E}_j, e_j)$ as shown in Fig. 20.

Note that all *k*-colored, *k*-sized subsets of vertices in graph *G* are represented by a world in model $$\mathcal {M}^{k+\left( {\begin{array}{c}k\\ 2\end{array}}\right) } = (\mathcal {M}, w_0) \otimes (\mathcal {E}_1, e_1) \otimes \cdots \otimes (\mathcal {E}_{k+\left( {\begin{array}{c}k\\ 2\end{array}}\right) }, e_{k+\left( {\begin{array}{c}k\\ 2\end{array}}\right) })$$, and each world in $$\mathcal {M}^{k+\left( {\begin{array}{c}k\\ 2\end{array}}\right) }$$ represents a *k*-colored, *k*-sized subset of vertices in graph *G*. Furthermore, some propositional variable $\psi _l=r_{ij}$ is true in world *w* in $$\mathcal {M}^{k+\left( {\begin{array}{c}k\\ 2\end{array}}\right) }$$ only if $x'_l$ and $x''_l$ are true in *w* and $\{x'_l, x''_l\}$ is an edge between vertices $x'_l$ and $x''_l$ in *G*.

Now we let $\varphi = \bigwedge \nolimits _{1 \le i < j \le k} r_{ij}$. By construction, we have that $\varphi$ is true in a world *w* only if all the *k* vertices in the *k*-colored, *k*-sized subset of vertices in graph *G* that are represented by *w* are connected by an edge in graph *G*. And conversely, if there is a world *w* corresponding to a set of *k* vertices with different colors that are all connected to each other by an edge in graph *G*, then in this world all propositional variables $r_{ij}$ are true, and thus $\varphi$ is true in *w*. Hence, $(G,c) \in \{k\}\text {-{Multicolored Clique}}{}$ if and only if $$\mathcal {M}^{k+\left( {\begin{array}{c}k\\ 2\end{array}}\right) } \models \varphi $$.

Since this reduction runs in polynomial time, parameters *a*, *c*, and *o* have a constant value, and parameters *f* and *u* depend only on parameter *k* (namely $$f = 2\left( {\begin{array}{c}k\\ 2\end{array}}\right) -1$$ and $$u = k + \left( {\begin{array}{c}k\\ 2\end{array}}\right) $$), we can conclude that $\{a,c,f,o,u\}$-DBU is W[1]-hard. $\square$

**Fig. 21:**
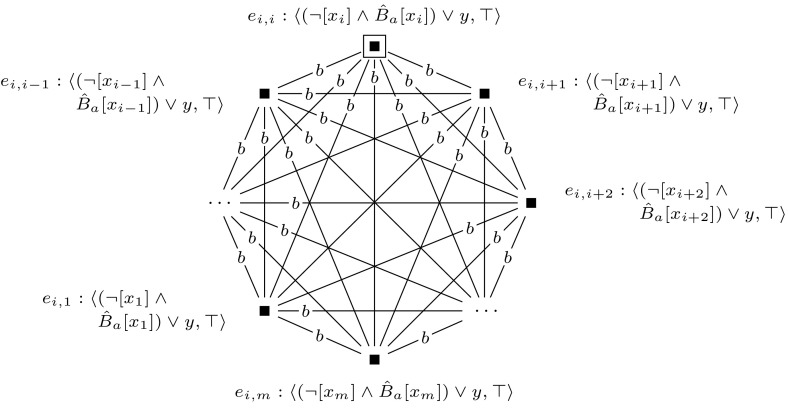
The event model  $(\mathcal {E}_i, e_{i,i})$, used in the proof of Proposition 5

##### Proposition 5

$$\{c,o,p,u\}\text {-DBU}$$ is W[1]-hard.

##### Proof

To show W[1]-hardness, we specify an fpt-reduction *R* from $\{k\}$-WSat[2CNF] to $\{c,o,p,u\}\text {-DBU}$. Let $\varphi$ be a propositional formula with *var*$(\varphi ) = \{x_1, \ldots , x_m\}$. Without loss of generality, assume that $m \ge 2$. We introduce agent *b* and construct a single-pointed epistemic model $(\mathcal {M}, w_0)$, an applicable sequence of single-pointed event models $(\mathcal {E}_1, e_1), \ldots , (\mathcal {E}_k, e_k)$, and a formula $[\varphi ] \in \mathcal {L}_B$, such that $\varphi \in \{k\}-{WSat}\text {[2CNF]}{}$ if and only if $(\mathcal {M}, w_0) \otimes (\mathcal {E}_1, e_1) \otimes \cdots \otimes (\mathcal {E}_k, e_k) \models \hat{B}_b [\varphi ']$.

We sketch the general idea behind the reduction. Let $\varphi$ be a propositional formula with *var*$(\varphi ) = \{x_1, \ldots , x_m\}$. Then let $\varphi '$ be the formula obtained from $\varphi$ by replacing each occurrence of $x_i$ with $\lnot x_i$. We note that $\varphi$ is satisfiable by some assignment $\alpha$ that sets *k* variables to true if and only if $\varphi '$ is satisfiable by some assignment $\alpha '$ that sets *k* variables to false, i.e, that sets $m-k$ variables to true. We use the reduction to list all possible assignments to *var*$(\varphi ')$ = *var*$(\varphi )$ that set $m - k$ variables to true. We represent each possible assignment to *var*$(\varphi ')$ that sets $m - k$ variables to true by a group of worlds, like in the proof of Theorem 1.

Just as in the proof of Theorem 1, we construct a single-pointed epistemic model $(\mathcal {M},w_0)$ with agents $a, 1, \ldots , m$ and propositional variable *y*, as shown in Fig. 6. Then for each $1 \le i \le k$, we define $(\mathcal {E}_i, e_{i,i})$ as shown in Fig. 21. Note that in each event model the designated event is $e_{i,i}$. This is to ensure that the event models are applicable. Now model $\mathcal {M}^k = (\mathcal {M}, w_0) \otimes (\mathcal {E}_1, e_1) \otimes \cdots \otimes (\mathcal {E}_k, e_k)$ consists of groups of worlds that each represent some assignment $\alpha '$ to *var*$(\varphi ')$ = *var*$(\varphi )$ that sets $m-k$ variables to true. Each assignment $\alpha$ to *var*$(\varphi ')$ = *var*$(\varphi )$ that sets $m-k$ variables to true is represented by a group of worlds in model $\mathcal {M}^k$.

For $1\le i \le m$, let $[x_i] = \hat{B}_i y$. Now we let $[\varphi ]$ be the adaptation of formula $\varphi$ where every occurrence of $x_i$ in $\varphi$ is replaced by $\hat{B}_a[x_i]$. By construction, formula $\hat{B}_b [\varphi ']$ is true in model $\mathcal {M}^k$ if and only if there is a group of worlds in $\mathcal {M}^k$ that represents some assignment $\alpha '$ that sets $m-k$ variables to true and that satisfies formula $\varphi '$, i.e., if and only if there is some assignment $\alpha$ that satisfies formula $\varphi$ and that sets *k* variables to true. Hence, $(\varphi , k) \in$ $\{k\}$-WSat[2CNF] if and only if $(\mathcal {M}, w_0) \otimes (\mathcal {E}_1, e_1) \otimes \cdots \otimes (\mathcal {E}_k, e_k) \models \hat{B}_b [\varphi ']$.

Since this reduction runs in polynomial time, parameters *c*, *o*, and *p* have constant values, and parameter *u* depends only on parameter *k* (namely $u = k$), we can conclude that $\{c,o,p,u\}\text {-DBU}$ is W[1]-hard. $\square$

**Fig. 22:**
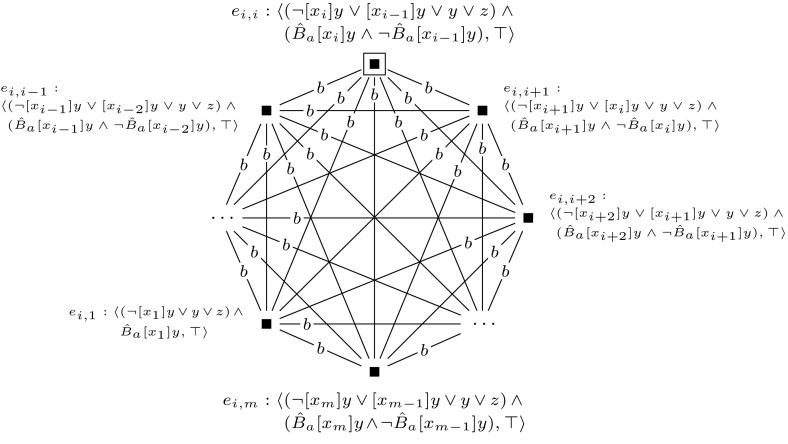
The event model $(\mathcal {E}_i, e_{i,i})$, used in the proof of Proposition 6

##### Proposition 6

$$\{a,f,o,p,u\}\text {-DBU}$$ is W[1]-hard.

##### Proof

To show W[1]-hardness, we specify an fpt-reduction *R* from $\{k\}$-WSat[2CNF] to $\{a,f,o,p,u\}\text {-DBU}$. Let $\varphi$ be a propositional formula with *var*$(\varphi ) = \{x_1, \ldots , x_m\}$. Without loss of generality, assume that $m \ge 2$. We introduce propositional variable $z^*$ and construct a single-pointed epistemic model $(\mathcal {M}, w_0)$ and an applicable sequence of single-pointed event models $(\mathcal {E}_1, e_1), \ldots , (\mathcal {E}_{k+1}, e_{k+1})$, such that $\varphi \in \{k\}-{WSat}\text {[2CNF]}{}$ if and only if $(\mathcal {M}, w_0) \otimes (\mathcal {E}_1, e_1) \otimes \cdots \otimes (\mathcal {E}_{k+1}, e_{k+1}) \models z^*$.

We modify the reduction in the proof of Proposition 5 to keep the values of parameters *a* and *f* constant. To keep the number of agents constant, we use the same strategy as in the reduction in the proof of Proposition 3, where variables $x_i,\ldots ,x_m$ are represented by strings of worlds with alternating relations $R_b$ and $R_a$. And just like in the proof of Proposition 3, the size of the formula is kept constant by encoding the satisfiability of the formula with a single propositional variable. Then each group of worlds that represents a satisfying assignment for the given formula will have an $R_c$ relation from a world that is $R_b$-reachable from the designated world to a world where propositional variable $z^*$ is true.

We introduce a single-pointed epistemic model $(\mathcal {M},w_0)$ with agents *a* and *b*, and propositional variables *y* and *z*, such as defined in Fig. 15. Let $[x_i]$ be defined as in the proof of Proposition 3. Then for each $1 \le i \le k$, we define $(\mathcal {E}_i, e_{i,i})$ as shown in Fig. 22. Again, to ensure that the event models are applicable, in each event model the designated event is $e_{i,i}$. Now model $\mathcal {M}^k = (\mathcal {M}, w_0) \otimes (\mathcal {E}_1, e_1) \otimes \cdots \otimes (\mathcal {E}_k, e_k)$ consists of groups of worlds that each represent some assignment $\alpha$ to *var*$(\varphi )$ that sets $m-k$ variables to true. And conversely, each assignment $\alpha$ to *var*$(\varphi )$ that sets $m-k$ variables to true is represented by a group of worlds in model $\mathcal {M}^k$.

**Fig. 23:**
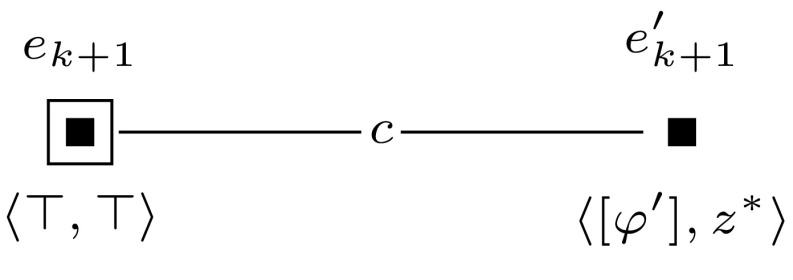
The event model $(\mathcal {E}_{k+1}, e_{k+1})$, used in the proof of Proposition 6

Let $\varphi '$ be the formula obtained from $\varphi$ by replacing each occurrence of $x_i$ with $\lnot x_i$, and let $[\varphi ]$ be defined as in the proof of Proposition 3. We introduce agent *c* and define event model $(\mathcal {E}_{k+1}, e_{k+1})$ as shown in Fig. 23. Event model $(\mathcal {E}_{k+1}, e_{k+1})$ makes sure that each group of worlds that represents a satisfying assignment for formula $\varphi '$ will be marked by propositional variable $z^*$. Thereby the size of formula *f* is kept constant. Now, in a similar way as in the proof of Proposition 5, we have that  $(\varphi , k) \in$ $\{k\}$-WSat[2CNF] if and only if $(\mathcal {M}, w_0) \otimes (\mathcal {E}_1, e_1) \otimes \cdots \otimes (\mathcal {E}_k, e_k) \models z^*$.

Since this reduction runs in polynomial time, parameters *a*, *f*, *o*, and *p* have constant values, and parameter *u* depends only on parameter *k* (namely $u = k+1$), we can conclude that $\{a,f,o,p,u\}\text {-DBU}$ is W[1]-hard. $\square$

#### Fixed-Parameter Tractability Results

Next, we turn to a case that is fixed-parameter tractable.

##### Theorem 3

$\{e,u\}$-DBU is fixed-parameter tractable.

##### Proof

We present the following fpt-algorithm that runs in time $e^u \cdot p(| x|)$, for some polynomial *p*, where *e* is the maximum number of events in the event models and *u* is the number of event models (i.e., the number of updates).

As a subroutine, the algorithm checks whether a given basic epistemic formula $\varphi$ holds in a given pointed epistemic model $(\mathcal {M},W_d)$, i.e., whether $\mathcal {M},W_d \models \varphi$. This can be done in polynomial time in the size of $\mathcal {M}$ plus the size of $\varphi$. First, we note that for every modal logic formula $\varphi$, we can construct in polynomial time a first-order logic formula $\psi$ with two variables, such that checking whether $\varphi$ is true in a given pointed epistemic model $(\mathcal {M}, W_d)$ can be done by checking the truth of $\psi$ in $\mathcal {M}$. We can construct this $\psi$ by means of the standard translation (van Benthem 1977, Definition 2.1), whose definition can straightforwardly be adapted to the case of multiple agents. This adapted definition can also be used for the slightly more general case of multi-pointed models.

Furthermore, given a model and a formula in first-order logic with a constant number of variables, checking whether the formula is true in the model can be done in polynomial time in the size of the model plus the size of the formula (Vardi 1995, Proposition 3.1). Therefore, we can decide the truth of a given modal logic formula in a given model in polynomial time.

Let $x = (P, \mathcal {A}, (\mathcal {M}_0, w_0), (\mathcal {E}_1, e_1), \ldots , (\mathcal {E}_u, e_u), \varphi )$ be an instance of DBU. First the algorithm computes the final updated model $M^m= (\mathcal {M}_0, w_0) \otimes (\mathcal {E}_1, e_1) \otimes \dots \otimes (\mathcal {E}_u, e_u)$ by sequentially performing the updates. Let $\mathcal {M}^0 = (\mathcal {M}_0, w_0)$, then for each *i*, $\mathcal {M}^i$ is defined as $\mathcal {M}^{i-1} \otimes (\mathcal {E}_i, e_i)$. The size (i.e., the number of events) of each $\mathcal {M}^i$ is upper bounded by $|\mathcal {M}^0| \cdot e^u$, so for each update checking the preconditions can be done in time polynomial in $e^u \cdot |x|$ by using the subroutine we described above. This means that computing $\mathcal {M}^u$ can be done in fpt-time.

Then the algorithm decides whether $\varphi$ is true in $\mathcal {M}^u$. This can be done in time polynomial in the size of $\mathcal {M}^u$ plus the size of $\varphi$. We know that $|\mathcal {M}^u| + |\varphi |$ is upper bounded by $|\mathcal {M}^0| \cdot e^u + |\varphi |$ and thus upper bounded by $e^u \cdot p(|x|)$ for some polynomial *p*. Therefore, deciding whether $\varphi$ is true in $\mathcal {M}^u$ is fixed-parameter tractable. Hence, the algorithm decides whether $x \in$ DBU and runs in fpt-time. $\square$

### Overview of the Results

**Fig. 24:**
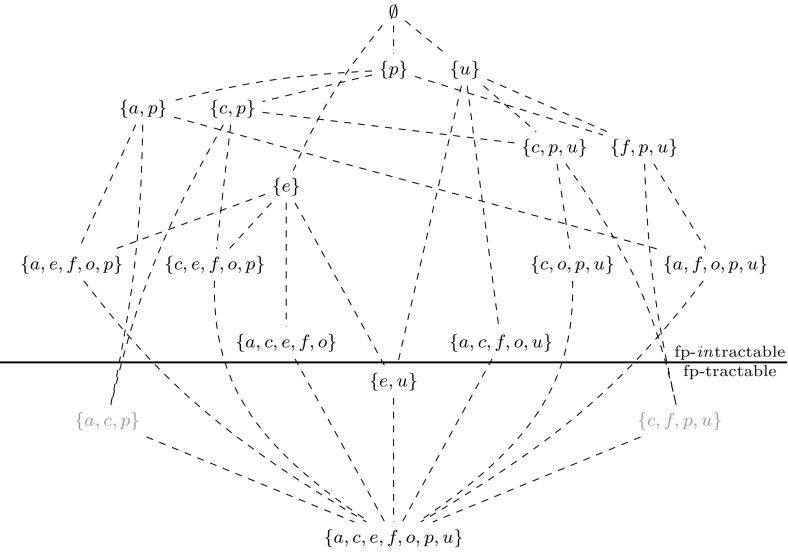
Overview of the parameterized complexity results for the different parameterizations of DBU, and the line between fp-tractability and fp-intractability. For the readability of this illustration, this graph describes the case where $\{a,c,p \}$-DBU and $\{c,f,p,u \}$-DBU are fp-tractable, which we leave as open problems

We showed that DBU is $\text { PSPACE}$-complete, we presented several parameterized intractability results (W[1]-hardness and para-NP-hardness) and we presented one fixed-parameter tractability result, namely for $\{e,u\}$-DBU. In Fig. 24, we present a graphical overview of our results and the consequent border between fp-tractability and fp-intractability for the problem DBU. We leave $\{a,c,p\}\text {-DBU}$ and $\{c,f,p,u\}\text {-DBU}$ as open problems for future research.

## Discussion

Theory of mind reasoning is notorious in the cognitive science literature for its presumed computational intractability. A possible reason could be that it may involve higher-order thinking (e.g., ‘you believe that I believe that you believe’). To investigate this we formalized theory of mind reasoning as updating of beliefs about beliefs using dynamic epistemic logic (DEL). We presented the Dynamic Belief Update (DBU) model (which is a special case of DEL model checking), and we proved that it is PSPACE-complete. This means that (without additional constraints), there is no algorithm that computes DBU in a reasonable amount of time. In other words, without restrictions on its input domain, the model is computationally too hard to serve as a plausible explanation for human cognition. To investigate whether the ‘order parameter’ is a source of intractability, i.e., whether restricting the order of reasoning could render DBU tractable, we analyzed the parameterized complexity of DBU.

Possibly counterintuitively from a cognitive science perspective, we did not find any fixed-parameter tractability results for the order parameter, which we operationalized as the modal depth of formula $\varphi$ in DBU. Already for a formula with modal depth one (even with just a single agent) DBU is NP-hard. It follows as a corollary from Proposition 1 that $\{o\}$-DBU (and $\{a\}$-DBU, and $\{a,o\}$-DBU) are para-NP-hard. For the parameters that we considered, we did not find any set of parameters for which adding order parameter *o* makes DBU fixed-parameter tractable. Note that we left $\{a,c,p\}\text {-DBU}$ and $\{c,f,p,u\}\text {-DBU}$ as open problems. If $\{a,c,p\}\text {-DBU}$ or $\{c,f,p,u\}\text {-DBU}$ turn out to be fixed-parameter intractable, then there could be cases for which adding order parameter *o* makes a difference; e.g., $\{a,c,o,p\}$-DBU or $\{c,f,o,p,u\}$-DBU could then be fixed-parameter tractable. In these cases, only in combination with at least three or four other parameters could restricting the order parameter lead to fixed-parameter tractability. Summing up, we found no evidence to support the claim that higher-order thinking is a source of intractability for theory of mind. It is important not to confuse computational intractability with cognitive difficulty here. Higher-order thinking could still be a source of difficulty for performing theory of mind for other reasons than computational complexity (e.g., due to limited working-memory).

It is interesting to note that in our $\text { PSPACE}$-hardness proof we used more than one agent, while for DBU restricted to a single agent we were only able to show NP-hardness. It is known that for games the complexity of the single-agent case often lays low in the polynomial hierarchy (e.g., NP-complete), while the two-agent case usually brings the complexity up to $\text { PSPACE}$-completeness (see, e.g., Burke et al. 2015; Reyzin 2006). We find this remarkable and we expect the same to hold for DBU. Determining the exact upper bound on the complexity of DBU restricted to a single agent, we leave for future research. Just like the modal depth, the number of agents seems to be related to higher-order theory of mind. Restricting a problem to a single agent, basically means eliminating the possibility of higher-order theory of mind. Allowing for two or more agents, on the other hand, means that there can be agents reasoning about each other; i.e., it allows for higher-order theory of mind. Like for many games, restricting DBU to a single agent does not render it tractable, as already for a single agent it is NP-hard. It is remarkable that even though it does not make the difference between tractability and intractability, the difference between one or more agents might still make the difference between a lower or higher location in the complexity landscape.

While we did not find any tractability results for the order parameter, we did find a fixed-parameter tractability result for the combination of two other parameters that we considered—namely, for parameters *e* and *u*, respectively the maximum number of events in any of the given event models and the number of event models, (i.e., the number of updates). When restricting only the number of events in the event models or only the number of event models, this does not render DBU tractable, but restricting both does. This shows that *e* and *u* together form a source of intractability. This result is perhaps not surprising since these two parameters determine the size of the search space for DBU. This is because the size of the final updated model $(\mathcal {M}_0, W_d) \otimes (\mathcal {E}_1, E_1) \otimes \cdots \otimes (\mathcal {E}_u, E_u)$ (i.e., the search space for DBU) can be as large as and is upper bounded by the size of the initial model $(\mathcal {M}_0, w_0)$ times $e^u$. This fixed-parameter tractability result is consistent with the fact that a sequence of event models $(\mathcal {E}_1, E_1), \ldots , (\mathcal {E}_u, E_u)$ can be combined into one event model $(\mathcal {E}', E')$ that has the same effect—at the cost of increasing the number of events to $e^u$ (van Ditmarsch and Kooi 2006). This means that combining the individual event models into one big event model does not buy tractability, since the size of the updated model $(\mathcal {M}_0, W_d) \otimes (\mathcal {E}', E')$ will still be as large as the size of the initial model $(\mathcal {M}_0, w_0)$ times $e^u$.

The question arises how we can interpret parameters *e* and *u* in terms of their cognitive counterparts. To what aspect of theory of mind do they correspond, and moreover, can we assume that they have small values in (many) real-life situations? In general, one could say that event models represent steps of change in time, and that the number of events in these event models represent the number of possible things (both factual and observational) that can happen. For instance, in the Sally-Anne example that we modeled the event model contains two events: one for the possibility that the marble stayed in the basket and one for the possibility that the marble was moved into the box while Sally is not aware of this. This event model stands for the change step in which ‘Sally leaves the room and Anne moves the marble into the box.’ It seems like a reasonable assumption that when considering situations of change, we do not consider many different steps of change at the same time, or that we consider many different things that could possibly happen in that situation. Of course, verifying this requires empirical research.

For the understanding of the complexity of theory of mind it is interesting that parameters *e* and *u* are unrelated to the order parameter. This seems to indicate that what makes theory of mind intractable is not necessarily higher-order thinking about others’ mental states but, more in general, reasoning about change. So our results indicate that, contrarily to what is often believed, the intractability of theory of mind is not due to involvement of a specialized form of reasoning for the social domain.

Besides the implications for the understanding of the complexity of theory of mind, our complexity results might also be of independent interest for researchers working on dynamic epistemic logic. Previous results have shown that DEL model checking is $\text { PSPACE}$-complete for the case restricted to single-pointed KD45 models (Aucher and Schwarzentruber 2013; Bolander et al. 2015; van Eijck and Schwarzentruber 2014). We showed that DEL model checking is $\text { PSPACE}$-complete even for the more restricted case of single-pointed S5 models. We also analyzed the problem from a parameterized complexity point of view. We proved that for most combinations of the considered parameters the problem is fixed-parameter intractable (by means of hardness results for the classes W[1] and para-NP), and for one case we proved fixed-parameter tractability (see Fig. 24 for an overview). These parameterized complexity results can be informative for researchers interested in implementations of DEL in computer science and AI. Understanding which parameters are sources of intractability in DEL-based models of epistemic reasoning informs such applied research efforts by specifying under which conditions tractable belief updating is possible and when it is not.

## Conclusion

We analyzed the computational complexity of theory of mind, formalized as the updating of beliefs about beliefs in dynamic epistemic logic (DEL). A key finding is that our results suggest that the intractability of theory of mind is not due to the computational demands imposed by ‘higher-order reasoning,’ as often assumed in cognitive science. Instead, our results suggest that intractability of theory of mind may be better sought in the computational demands posed by a more general form of reasoning about steps of change in time.

In sum, our work showcases how logic and complexity theory can inform debates in cognitive science. We adopted this methodological approach and conceptual framework to help bridge the areas of logic and cognitive science. We hope that it may serve as a guide for more future research at the interface of logic, complexity, and cognition. Future research may include investigating alternative ways to parameterize higher-order thinking in DEL. It would be interesting to see whether there are other ways to formalize and possibly bound the order of reasoning in DEL models. For a broader applicability of using DEL for modeling theory of mind, future research may furthermore include extending the model to a wider palette of mental states, like emotional and motivational states.

## Footnotes

1 A note about terminology—throughout this paper we will use the term ‘theory of mind.’ In some literature, instead of theory of mind, the term mindreading is used to refer to the same cognitive capacity. Note that theory of mind is not actually a ‘theory’ but refers to a cognitive capacity. To emphasize the fact that theory of mind is a capacity rather than a theory, we will also refer to it as ‘theory of mind reasoning,’ or as the ‘theory of mind capacity.’

2 In the most recent poll by Gasarch (2012) an overwelming majority of computer scientists thinks that $\text { P}\ne \text { NP}$.
